# Development of a laser injury model using human cardiac spheroids

**DOI:** 10.1242/dmm.052855

**Published:** 2026-10-28

**Authors:** Asli Adak, Romario Strasser, Marco Meer, Prateek Arora, Marco Osterwalder, Nadia Mercader, Christian Zuppinger

**Affiliations:** ^1^ Institute of Anatomy, University of Bern, 3012 Bern, Switzerland; ^2^ Department for BioMedical Research (DBMR), University of Bern, 3008 Bern, Switzerland; ^3^ Graduate School for Cellular and Biomedical Sciences (GCB), University of Bern, 3012 Bern, Switzerland; ^4^ Department of Cardiology, Inselspital, 3010 Bern, Switzerland; ^5^ Centro Nacional de Investigaciones Cardiovasculares Carlos III (CNIC), 28029 Madrid, Spain

**Keywords:** Laser ablation, Microtissue, Spheroid, Injury, Cardiac regeneration, Fibrosis

## Abstract

Myocardial infarction is a leading cause of death and leads to irreversible loss of cardiomyocytes and the deposition of a fibrotic scar. Here, we developed a human cardiac microtissue model that enables controlled induction of focal laser injury to model cardiac injury-induced cellular responses. This system is based on self-assembly of troponin-I1-EGFP-positive human induced pluripotent stem cell-derived cardiomyocytes (iPSC-CMs) and cardiac fibroblasts (iPSC-CFs) into contracting spheroids. Targeted UV-laser ablation induced a localized injury affecting nearly 30% of the spheroid area and led to both necrosis and cardiomyocyte apoptosis at the injury site. The procedure transiently reduced contractility, which recovered after 1 week of culture. Laser injury did not stimulate cell proliferation, but evoked a limited fibrotic response, evidenced by proteomics and immunostaining of cryosections and cleared spheroid preparations. Our findings establish cardiac spheroids as a human *in vitro* model to study the effects of cardiac injury on a cardiomyocyte and fibroblast co-culture system, with the potential to study cell interactions and screen for compounds promoting reparative or regenerative processes.

## Teaser

**Summary:** We report the establishment of a human cardiac microtissue injury model using focal laser ablation to investigate cardiomyocyte–fibroblast interactions during reparative and regenerative processes following cardiac injury.

## INTRODUCTION

Cardiac injury is often caused by myocardial infarction (MI), leading to loss of cardiomyocytes (CMs) and the formation of a fibrotic scar by activated cardiac fibroblasts (Cipriano et al., 2025; Prabhu and Frangogiannis, 2016). Despite the observed irreversible CM loss in adult tissue, a few case reports suggest that newborn children can recover after MI (Haubner et al., 2016). More recent studies suggest that the regenerative potential of the adult myocardium has been underestimated in the past, and patients with heart failure receiving left ventricle support device therapy had a >6-fold increase in CM renewal relative to that in the healthy heart (Derks et al., 2025). Indeed, in the mouse, the existence of neonatal heart regeneration is well established and shares similarities to that in other vertebrate models of high regenerative capacity, such as zebrafish (Tzahor and Poss, 2017). A fibrotic response precedes myocardial regeneration, as in both mouse and zebrafish (González-Rosa et al., 2011), and current research points to a role of specific fibroblasts in promoting CM proliferation and myocardial regeneration (Hu et al., 2022; Sánchez-Iranzo et al., 2018). Thus, understanding how fibroblasts interact with CMs in the context of an injury response, and which pathways drive repair versus regeneration, is of great interest.

In summary, regenerative capacity is partially conserved in humans and mice but suppressed during the maturation of the heart. Although it is informative to understand the mechanisms of natural organ regeneration in animal models, it is of utmost importance to investigate whether any of the underlying genetic mechanisms could be employed to induce a regenerative effect in humans. For the study of these mechanisms *in vitro*, human cardiac model systems are necessary. However, healthy primary human CMs cannot be obtained from human tissue in sufficient quantities for research purposes. Human induced pluripotent stem cells (iPSCs) are more often used in biomedical research, as somatic cells from adult individuals, and patients with known medical history, can be reprogrammed into iPSCs (Yu et al., 2007). iPSCs can be expanded almost without limit, are amenable to precise genetic engineering and have the capacity to differentiate into diverse organotypic cell types (Cerneckis et al., 2024). CMs derived from human iPSCs (hiPSC-CMs) offer an efficient *in vitro* approach to model myocardial tissue properties, and several methods for their differentiation and maturation have been published (Lian et al., 2013). Protocols for the differentiation of hiPSC-derived cardiac fibroblasts (hiPSC-CFs) and other cardiovascular cell types are also available (Zhang et al., 2019).

Cardiac 3D culture can be achieved in multiple ways (Mohr et al., 2022; Zuppinger, 2019). Hydrogel-based microtissues, also known as engineered heart tissues (EHTs), are created by mixing cells with extracellular matrix (ECM) proteins, such as collagen or fibrin, and assume the shape of suspended bars or strings (Zimmermann et al., 2004). These microtissues can be electrically and mechanically stimulated, providing the force of the muscle construct as a readout (Eschenhagen et al., 1997). Cardioids represent another type of stem cell based, self-organizing organoids that recapitulate chamber-specific aspects of cardiac development in culture (Hofbauer et al., 2021). The approach we used here involves spheroids from human iPSCs pre-differentiated into cardiac iPSC-CFs and iPSC-CMs. A key advantage of these cardiac microtissues is that they can be assembled from one or several cardiac cell types by self-aggregation and are, therefore, scaffold free (Giacomelli et al., 2017; Kelm et al., 2004). Furthermore, by using spheroids, the number, size, composition and environment of the microtissue can be precisely controlled, resulting in a uniform population (de Korte et al., 2025). The small size of spheroids requires fewer cells per microtissue, which is an advantage over other 3D cell culture systems for screening projects.

Applications for the use of human 3D cardiac tissues include modeling MI, thereby providing insight into the mechanisms of cardiac repair and regeneration. Several cardiac injury methods have been deployed, including cryoinjury, induction of hypoxia combined with noradrenaline stimulation, photomanipulation, CoCl_2_ treatment followed by high glucose and calcium overload, and hypoxia/reoxygenation (Das et al., 2022; Hofbauer et al., 2021; Richards et al., 2020; Sharma et al., 2022; Veldhuizen et al., 2022; Voges et al., 2017; Zhang et al., 2025). Hydrogel-based microtissues subjected to cryoinjury showed cell death in the injury region, and, by 14 days post-injury, the tissues had regained spontaneous contractile function, with a trend toward increased cell proliferation (Voges et al., 2017). Another study using cardioids showed that cryoinjury led to an increase in CM necrosis at the injury zone, without altered proliferation capacities (Hofbauer et al., 2021). Das et al. (2022) demonstrated photomanipulation using focal laser injury applied to an EHT made of iPSC-CMs and primary human cardiac fibroblasts. This led to a fibrotic response in the focal injury zone and a transient reduction in contractile force, followed by recovery of function after 7 days. Findings from another study revealed that self-organizing cardiac organoids subjected to hypoxia-induced ischemia and ischemia–reperfusion exhibited cell death, functional impairments, changes in calcium handling, beating activity and enhanced collagen deposition (Song et al., 2024). Recently, a study by Zhang et al. (2025) reported that cardiac organoids subjected to hypoxia/reoxygenation using a hypoxic incubator exhibited apoptosis, oxidative stress, structural alterations and impaired electrophysiological function.

In contrast to the above-mentioned studies, we decided to use focal laser injury on comparably small cardiac microtissues, i.e. cardiac spheroids, composed of two specific cell types, to investigate their responses to focal injury, with the goal of specifically addressing the roles of iPSC-CMs and iPSC-CFs in reparative and regenerative mechanisms. This approach induced targeted cell death within a specific region, enabling us to investigate potential fibrotic and proliferative responses and to assess recovery of contractile function in a scalable human cardiac microtissue.

## RESULTS

### iPSC-derived CMs and CFs express expected marker proteins

Reagents and resources are listed in Table S11. Troponin-I1 (TNNI1)-EGFP-positive iPSCs and wild-type (WT)-iPSCs were differentiated into iPSC-CMs, and WT-iPSCs were differentiated into iPSC-CFs. In addition to the visual inspection of the iPSC-CMs and their spontaneous contractions, complete differentiation was corroborated by immunostaining with CM markers: iPSC-CMs expressed the M-line marker myomesin and Z-line marker sarcomeric α-actinin (Fig. S1Ai). Expression of EGFP-tagged TNNI1 and connexin 43 (also known as GJA1) was observed in iPSC-CMs (Fig. S1Aii). Ventricular and atrial myosin light chain-2 were both observed in iPSC-CMs (Fig. S1Aiii), indicating that the culture represented a mixed ventricular and atrial population, as previously observed (Zuppinger et al., 2017). During cardiac fibroblast differentiation, iPSCs undergo a transition from epicardial to epicardial-derived cell fate and finally adopt a cardiac fibroblast phenotype (Zhang et al., 2019). Stage-specific differentiation of iPSC-CFs was confirmed by immunostaining of epicardial progenitor cells for Wilms tumor protein 1 (WT1) and zona occludens protein-1 (ZO-1; also known as TJP1) at day 15 of differentiation (Fig. S1B). Fibroblast marker proteins DDR2, vimentin and collagen-1α-1 were detected in fully differentiated iPSC-CFs at day 21 (Fig. S1Ci-iii). Although α-smooth muscle actin (αSMA; also known as ACTA2) was expressed, it did not organize into stress fibers, consistent with a quiescent fibroblast phenotype rather than a myofibroblastic state (Pakshir et al., 2020). Spheroids made of 5000 cells reached an average diameter of 294±27 μm after 7 days and were beating at day 3 after seeding (Fig. 1B; Movie 1).

**Fig. 1.**
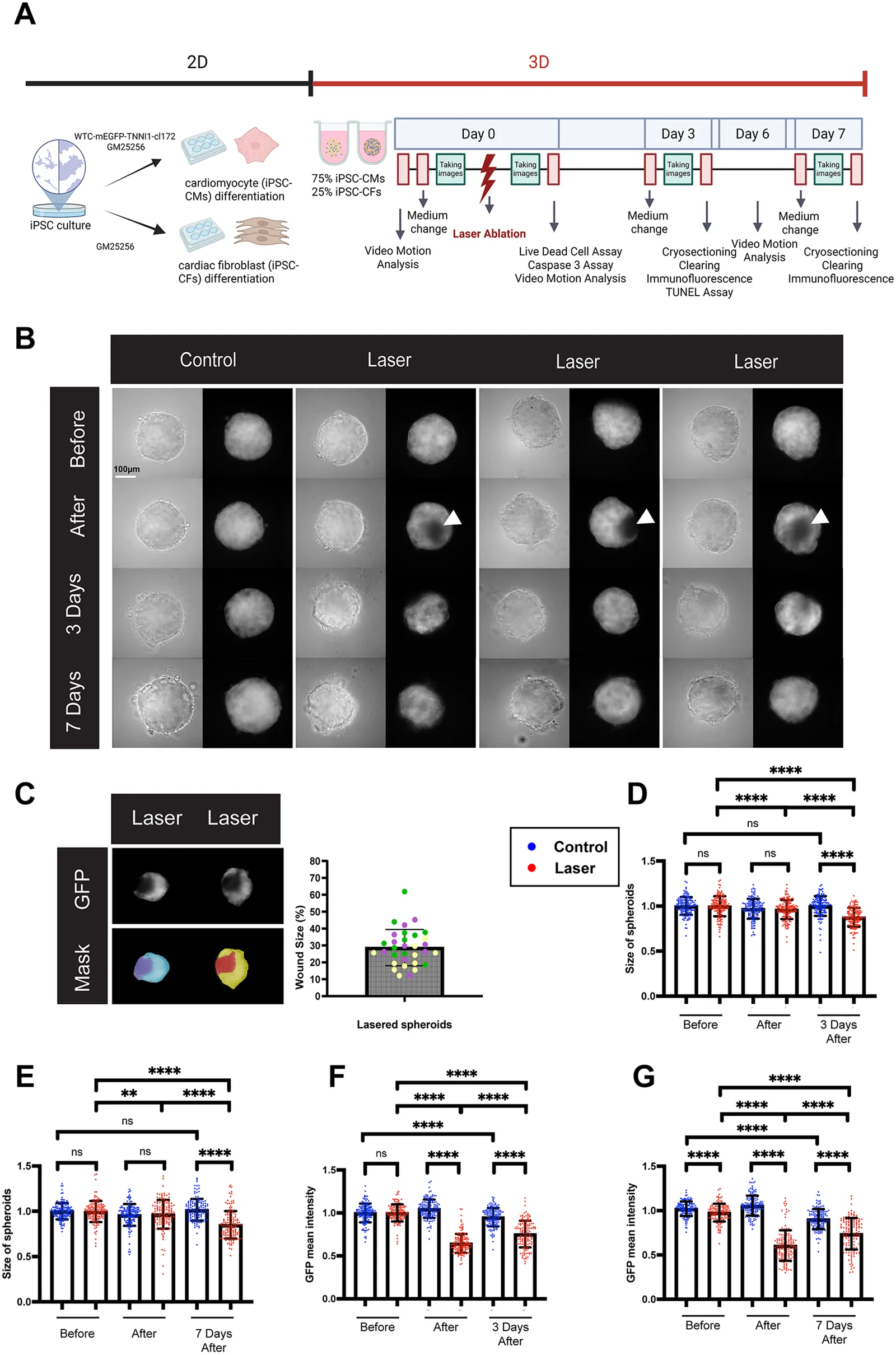
**Experimental timeline and effects of laser ablation on cardiac spheroids.** (A) Induced pluripotent stem cells (iPSCs) were initially cultured in a 2D format and differentiated into cardiomyocytes (CMs; iPSC-CMs) and cardiac fibroblasts (iPSC-CFs). Counted cells were then transferred to a 96-well culture plate coated with a non-adhesive coating. The scheme shows the experimental timeline and research steps performed in the days pre- and post-laser ablation. TUNEL, terminal deoxynucleotidyl transferase dUTP nick end labeling. Created in BioRender by Zuppinger, C. (2025). https://BioRender.com/ltyumuq. This figure was sublicensed under CC-BY 4.0 terms. (B) Representative bright-field and GFP images of three injured spheroids and one control spheroid acquired with the Acquifer imager. Shown are images at the time points before and immediately after laser ablation (for control, no ablation), as well as 3 and 7 days later. Distinct dark areas indicating the site of injury are marked by white arrowheads. Scale bar: 100 μm. (C) Left: representative GFP images and calculated masks for the analysis of wound size. Right: wound size as percentage of spheroid area. Colors represent independent experiments, and each dot corresponds to a single spheroid (three biological replicates, *n*=10-11 spheroids per experiment). (D-G) Quantification of spheroid size (D,E) and the relative GFP fluorescence (F,G) of lasered and control spheroids over the 3- and 7-day culture period (three biological replicates; for 3 days with overall 143 spheroids and for 7 days with overall 139 spheroids). The size and the GFP intensities were normalized to the average values of the control or laser groups before injury. Each dot represents a spheroid. Blue dots represent control, and red dots represent lasered spheroids. Data are shown as mean±s.d. Mann–Whitney and Wilcoxon matched-pairs signed rank test. ns, not significant; **P<0.01, ****P<0.0001. Complete biostatistical comparisons are presented in Table S1.

### Focal laser ablation reduces spheroid size and the intensity of TNNI1-EGFP fluorescence in the apparent wound location

To study how spheroids react to a lesion, we performed laser ablation and imaged them directly after ablation, at 3 days post-ablation (dpa) or 7 dpa (Fig. 1A). Imaging EGFP expression in spheroids allowed us to assess injury levels and changes in fluorescence over time. Immediately after ablation, the region subjected to laser treatment showed a distinct dark area indicating the site of injury (Fig. 1B, arrowheads). The wound size of the lasered spheroids was assessed using image segmentation (Fig. 1C, left), and the segmented regions were quantified to measure the percentage of the wound area (Fig. 1C, right). The average wound area was 29±11% of the total spheroid area (three biological replicates). Moreover, we observed that, following laser injury, spheroid size was reduced at day 3 to 88±14% (Fig. 1D; Tables S1 and S2), and at day 7 to 84±18% (Fig. 1E; Tables S1 and S3), of that of the control group at the same time point, whereas the spheroid size of the control group remained unchanged. This suggests that the reduction in size is caused by the laser injury rather than general spheroid shrinkage. We observed that, at 3 and 7 dpa, lasered spheroids were not only smaller than untreated spheroids cultured for the same time span, but also showed a lower mean GFP intensity. The intensity was reduced at 3 dpa (79±19%) (Fig. 1F; Tables S1 and S2) and did not recover at 7 dpa (82±22%), compared to that of the control group at the same time point (Fig. 1G; Tables S1 and S3).

### Laser ablation causes cell death through necrosis and apoptosis

To assess the cellular damage created by the laser, several assays were employed, beginning with testing for immediate markers of cell death (plasma membrane rupture, caspase activity), followed by a terminal deoxynucleotidyl transferase dUTP nick end labeling (TUNEL) assay for the evaluation of DNA fragmentation as a late-stage marker of apoptosis (Fig. 2A). Using a live-dead assay comprising ethidium homodimer-1 (EthD-1) and calcein-AM staining, we were able to confirm that laser ablation led to visible signs of necrosis immediately after damage (Fig. 2B). Overall, we observed a 4.3-fold drop in the ratio of calcein-AM to EthD-1 signal intensity after laser ablation (Fig. 2C). To determine whether cells in the spheroids are undergoing apoptosis, a caspase activation assay was conducted in live spheroids (Fig. 2D). Laser ablation induced significant caspase-3/7 activation. We found a 3.2-fold increase in caspase activity in injured spheroids compared to that in controls (Fig. 2E). Finally, TUNEL assay confirmed DNA damage and apoptotic pathway activation upon laser ablation (Fig. 2F). A 2.6-fold increase in TUNEL-positive fluorescence was observed in the spheroid sections 3 days after injury (Fig. 2G). Overall, the results confirmed that the reduction in GFP intensity was not only a sign of photobleaching but represented evidence for cell death as an induced cellular response to laser ablation. This finding was corroborated by immunostaining of cryosections that demonstrated loss of TNNI1-EGFP in ablated spheroids (Fig. 3A) and by immunostaining for the sarcomeric protein myomesin, where an area of lost GFP-reporter fluorescence colocalized with absence of the sarcomeric protein (Fig. 3B).

**Fig. 2.**
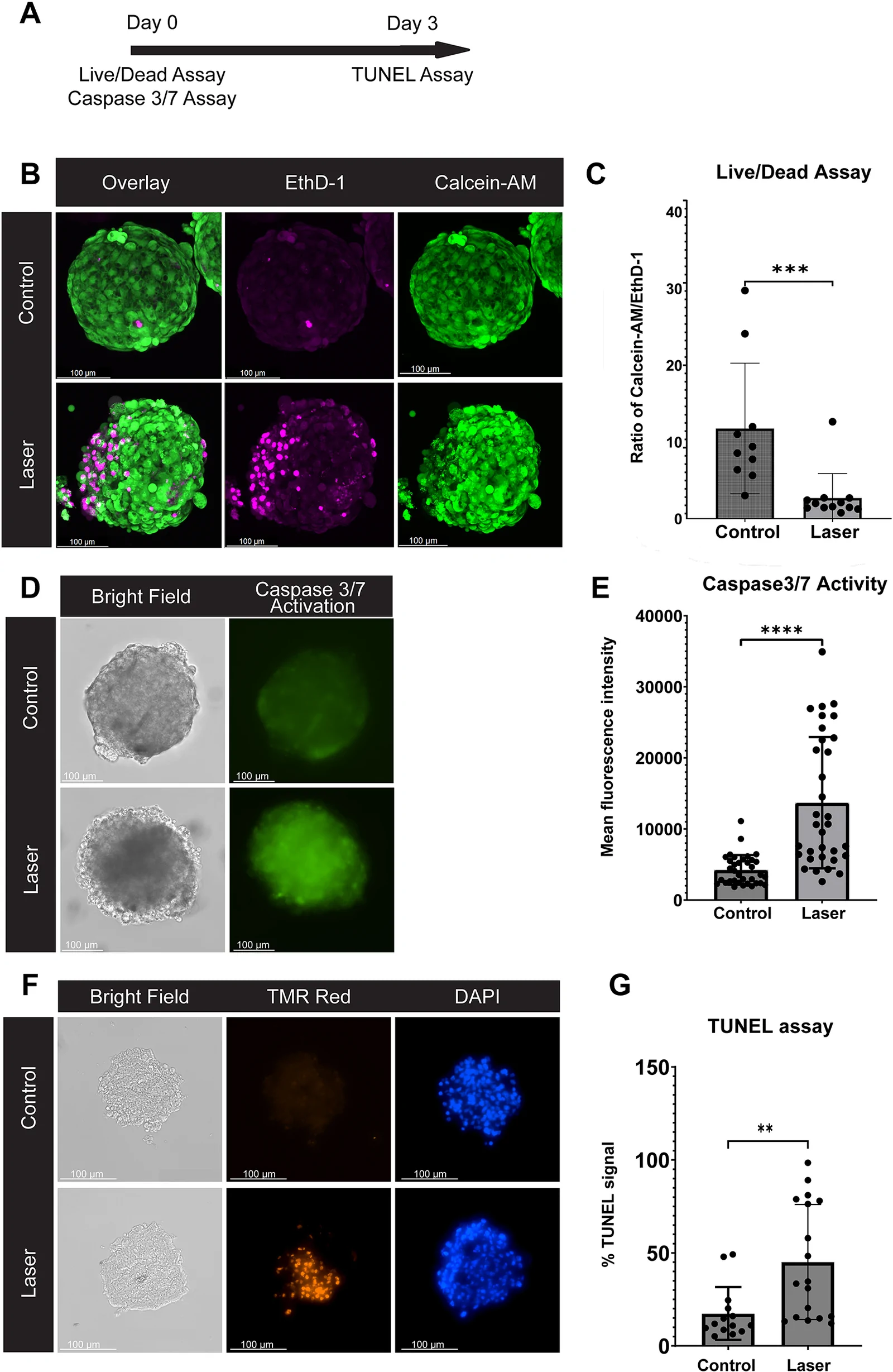
**Cell death assessment after laser ablation.** (A) Experimental timeline. (B) Single-channel and merged views of representative confocal images of live spheroids (maximum-intensity projection of 42 optical sections) stained with ethidium homodimer-1 (EthD-1; magenta) and calcein-AM (green). (C) Quantification of mean pixel intensities, plotted as a ratio of calcein-AM to EthD-1 (three biological replicates; control *n*=10, laser *n*=12). (D) Caspase-3/7 activation assay. Representative images of live spheroids in bright field and fluorescence using the FITC-filterset showing caspase-3/7 activity. (E) Quantification of FITC signal intensity (three biological replicates; control *n*=35, laser *n*=33). (F) TUNEL assay performed on spheroid cryosections 3 days after laser ablation (orange). Nuclei are counterstained with DAPI (blue). (G) Quantification of TUNEL signal intensity normalized to a positive DNAse treatment control (three biological replicates; control *n*=14, laser *n*=18). Mann–Whitney test. All graphs show individual spheroid measurements as well as mean±s.d. **P<0.01, ***P<0.001, ****P<0.0001. Scale bars: 100 μm.

**Fig. 3.**
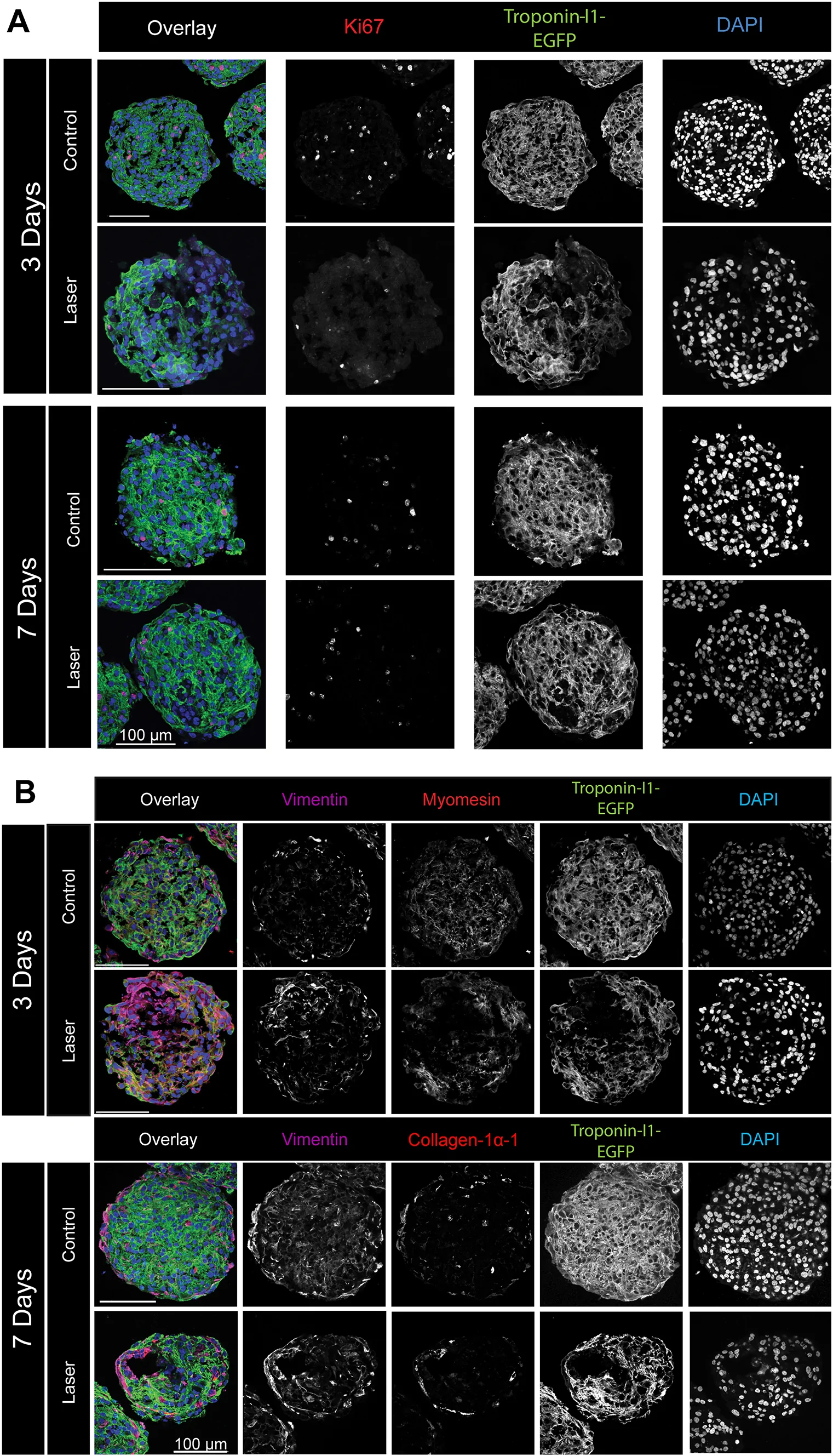
**Assessment of proliferation and fibrosis marker expression in cryosections.** Representative single and merged channel images of spheroid cryosections were immunostained and imaged using confocal microscopy. (A) Spheroid cryosections immunostained for Ki67 (red) and troponin-I1-EGFP (green) (*n*=3 technical replicates). Cell nuclei are counterstained with DAPI (blue). (B) Cryosections of spheroids were stained for vimentin (magenta), myomesin (red) and troponin-I1-EGFP (green) at the 3 days post-ablation (dpa) time point, and for vimentin (magenta), collagen-1α-1 (red) and troponin-I1-EGFP (green) at the 7 dpa time point. Nuclei are counterstained with DAPI (blue) (*n*=3 technical replicates). Scale bars: 100 μm.

### Cell proliferation is not increased in response to laser ablation

To study whether iPSC-CFs or iPSC-CMs expressing TNNI1-EGFP respond to focal laser injury by activating their cell cycle, we performed immunostaining with the cell proliferation marker Ki67 (also known as MKI67) (Arbustini et al., 1993) in whole-mount preparations and on cryosections at two time points after laser injury (Fig. 3A and Fig. 4A). Immunostaining against collagen-1α-1 to detect iPSC-CFs and GFP to detect TNNI1-EGFP-positive iPSC-CMs demonstrated that baseline proliferation was not limited to only one cell type (Fig. S2). As already observed using widefield fluorescence inspection (Fig. 1), immunostaining on cleared samples followed by confocal microscopy confirmed that laser-ablated spheroids exhibited a localized TNNI1-EGFP fluorescence-negative region, representing presumable injury regions that persisted at both 3 and 7 dpa (Fig. 4A, arrowhead in EGFP). However, qualitative assessment using Ki67 immunostaining did not indicate increased proliferation in laser-ablated spheroids at both 3 and 7 dpa compared to that in their respective control groups (Fig. 4A). Furthermore, TGFβ supplementation following laser ablation did not alter the number of Ki67-positive cells compared to those in lasered spheroids in control medium (Fig. S3E, Table S5). Counts of Ki67-positive nuclei per size of the respective layer (inner, middle, outer) were quantified using a 3D instance segmentation pipeline in whole-mount immunostained preparations (Fig. 4B,C; Fig. S4, Table S4).

**Fig. 4.**
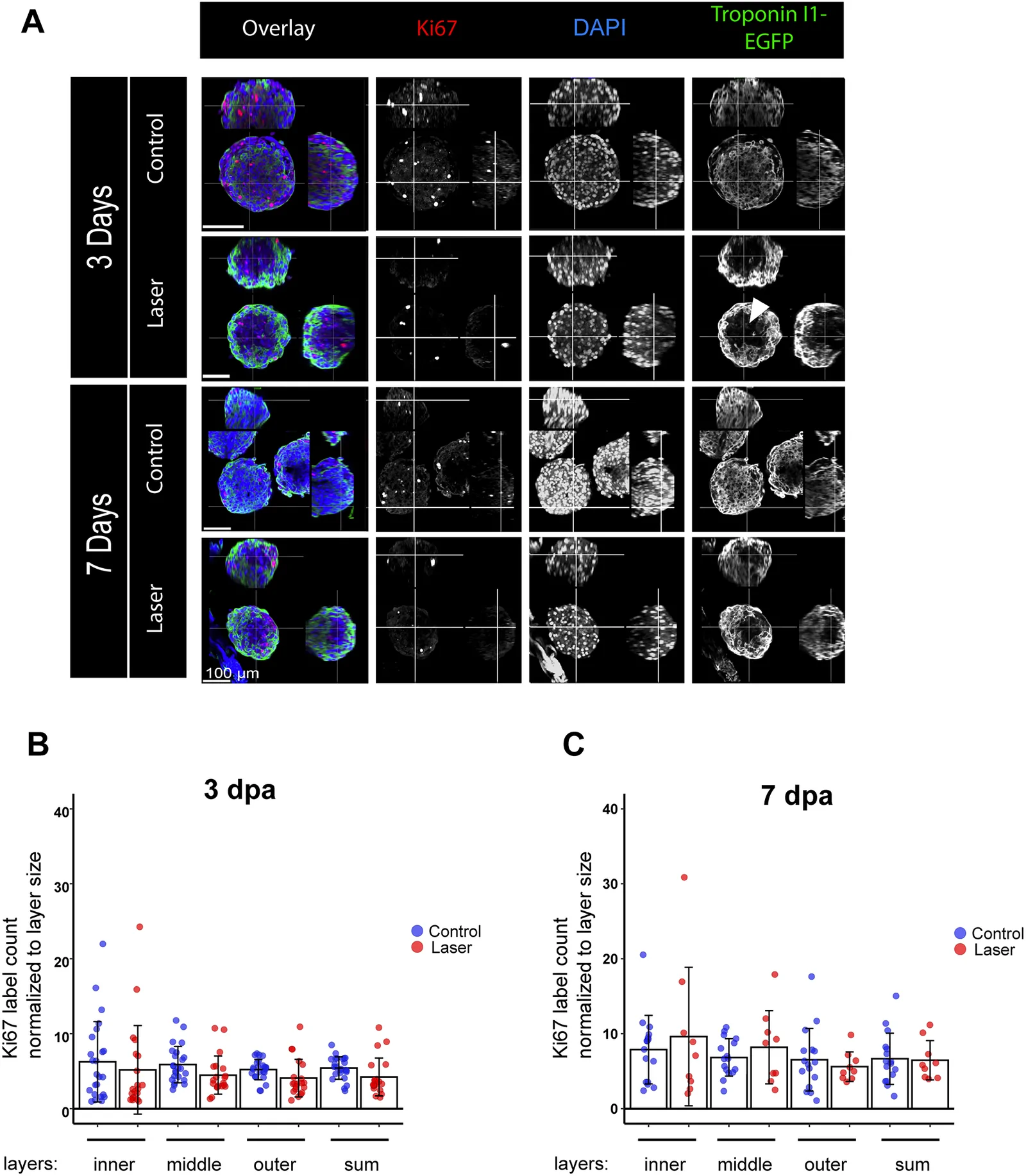
**Assessment of cell proliferation in lasered spheroids using whole-mount immunofluorescence.** (A) Representative confocal microscopy images of cleared whole-mount spheroids immunostained for Ki67 (red), and troponin-I1-EGFP (green). Nuclei are counterstained with DAPI (blue). Images in grayscale show a single optical section and respective orthogonal views of confocal image stacks. Arrowhead points to areas showing the absence of troponin-I1-EGFP. Scale bars: 100 μm. (B,C) Quantitative analysis of Ki67 label counts in spheroid layers (inner, middle, outer) normalized to the respective layer volume at 3 dpa (B) and 7 dpa (C). ‘Sum’ represents the total Ki67 label counts normalized to the entire half-spheroid volume (three biological replicates; control *n*=16, laser *n*=9). Kruskal–Wallis test. Shown are individual measurements as well as mean±s.d. Blue dots represent control, and red dots represent lasered spheroids.

In summary, Ki67 staining results suggest that, in response to laser injury, cell proliferation is not increased in CMs or the fibroblast population.

### Laser injury leads to the expression of fibrosis markers in spheroids

A hallmark of human cardiac injury response is the development of fibrosis (Prabhu and Frangogiannis, 2016). Immunostaining of cryosections for vimentin and collagen-1α-1 suggested the accumulation of vimentin and collagen-1α-1 signals upon laser-ablation (Fig. 3B). Qualitatively, vimentin and collagen signals were found to be enriched in regions exhibiting reduced TNNI1-EGFP signal at 3 and 7 dpa in cleared and immunostained spheroids (Fig. 5A). 3D quantitative analysis showed no significant difference in fibronectin expression at either 3 dpa or 7 dpa (Fig. 5B,E; Table S4). In contrast, vimentin expression showed a significant increase in the inner and middle layers at 3 dpa (Fig. 5C; Table S4), and a significant increase in the inner (4.5-fold) and middle layers (4.2-fold) at 7 dpa (Fig. 5F; Table S4). Collagen-1α-1 showed a significant increase at 3 dpa (Fig. 5D; Table S4) but not at 7 dpa (Fig. 5G; Table S4). Additionally, repeating the experiment in a different cell culture medium (lacking FGF2 and with higher levels of ascorbic acid) led to a significant increase in fibronectin signal at 7 dpa in lasered spheroids compared to that in untreated controls (Fig. S3). TGFβ supplementation further increased fibronectin signal and, additionally, led to an increment in vimentin and collagen-1α-1 signal detection (Fig. S3A-C). A complete differentiation into myofibroblasts was presumably only achieved upon TGFβ supplementation, as this condition increased αSMA levels compared to those in the laser-only condition (Fig. S3D; Table S5). Overall, our results show a mild fibrotic response in areas of the spheroids that have undergone cellular damage.

**Fig. 5.**
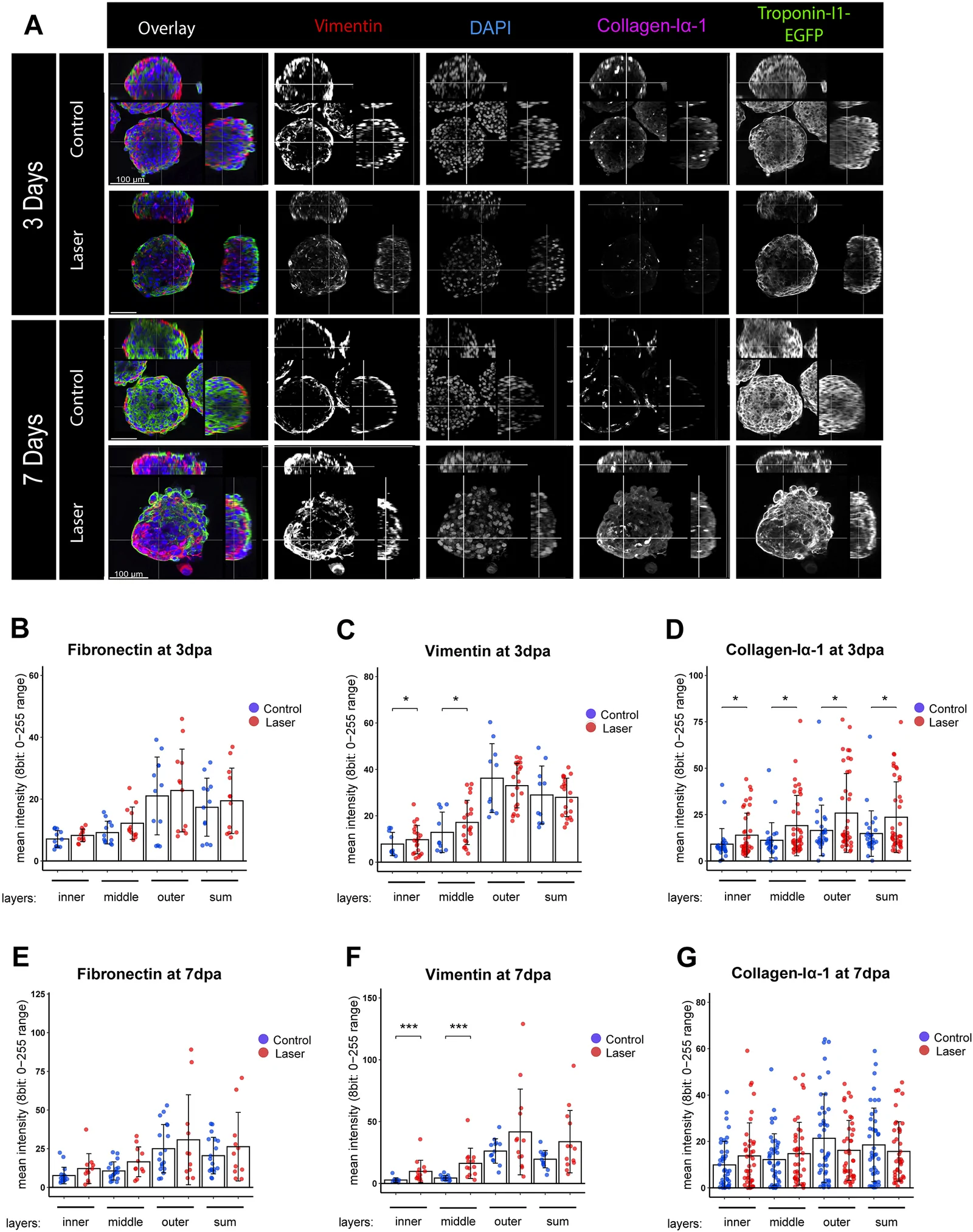
**Assessment of fibrosis in lasered spheroids using whole-mount immunofluorescent staining.** (A) Representative confocal images of cleared whole-mount spheroids immunostained for vimentin (red), collagen-1α-1 (magenta) and troponin-I1-EGFP (green). Nuclei are counterstained with DAPI (blue). Images in grayscale show a single optical section and respective orthogonal views of confocal image stacks. Scale bars: 100 μm. (B-G) Quantitative analysis of fibrosis marker mean intensity (8-bit: 0-255) within three spheroid layers (inner, middle, outer) at different time points. ‘Sum’ represents the mean marker intensity within the entire half-spheroid volume. Quantification of fibronectin at 3 dpa (B) and 7 dpa (E); vimentin at 3 dpa (C) and 7 dpa (F); and collagen-1α-1 staining mean intensities at 3 dpa (D) and 7 dpa (G). Three biological replicates; control *n*=6, laser *n*=9. Significance was assessed via mixed-effects models as described in the Materials and Methods (‘Statistical analysis’ section). Shown are individual measurements as well as mean±s.d. *P<0.05, ***P<0.001. Blue dots represent control, and red dots represent lasered spheroids.

### Proteomic profiling reveals a hemostatic, fibrotic and metabolic remodeling signature 7 days after laser ablation

To expand the characterization of the effects of laser ablation observed by immunostaining, we performed an unbiased proteomics analysis of the spheroids with and without laser injury at 7 dpa in medium lacking FGF2 and with higher levels of ascorbic acid. Of the 4080 identified proteins, we observed 409 to be differentially expressed at 7 dpa (Fig. 6; Table S6). Over-representation analysis of these proteins using the Gene Ontology Biological Pathways (GO-BP) as the reference (Tables S7 and S8) showed an upregulation of wound-healing/coagulation-associated proteins, i.e. F2, F5, F10, F13A1, plasminogen (PLG), serine proteinase inhibitor 1 (SERPINE1), bone morphogenetic protein-1 (BMP1) and vitronectin (VTN). Pathway analysis using the Gene Ontology Cellular Components (GO-CC) database (Tables S9 and S10) showed enrichment of proteins related to secretory granule/cytoplasmic vesicle/vesicle lumen, endoplasmic reticulum lumen and Golgi lumen, including upregulation, after laser injury, of F13A1, PLG, SERPINE1, PROS1, THBS1, F5, GRN, PAFAH1B2, FERMT3, F2, F10, SERPINC1, VTN, NUCB1 and FSTL1. This suggests that laser injury activates the secretion of wound healing/coagulation pathway proteins. We also observed upregulation of pro-fibrotic matricellular and ECM-remodeling responses using GO-BP [connective tissue growth factor (CCN2), THBS1, PLOD2 increased; MMP9 decreased]. This was corroborated in GO-CC, in which pathways such as ECM were upregulated [F13A1, F2, PLG, SERPINC1, VTN, SERPINE1, THBS1, CCN2, CRELD1 and growth/differentiation factor-15 (GDF15)]. Furthermore, proteomics suggested a metabolic shift from a glycolytic to an oxidative/mitochondrial metabolism. Oxidative phosphorylation-related proteins such as NDUFB1, NDUFB8, COX5A, COX5B, ATP5PD, ATP5F1D, NDUFV2, COQ9 and AK4 were upregulated, and glycolysis-related proteins including LDHA, GPI, ALDOC, ENO3, PGAM2, TPI1, GOT1 and MDH1 were downregulated, upon laser injury. Moreover, there was an increase in pro-survival PI3K/AKT-associated signaling proteins (IGF1R, PRKCA, GDF15) in lasered spheroids compared to those in controls. In addition to these pathways, two sarcomeric markers, TNNI1 and cardiac troponin T (TNNT2), were both significantly decreased in lasered spheroids, confirming persistent CM loss post-laser injury observed with cell death assays (Fig. 2). Moreover, several apoptosis-associated proteins (FADD, CASP7, CYCS, PARP1) and the necrosis-associated proteins HMGB1 and HMGB2 were decreased at this later time point, consistent with resolution of the acute cell-death response documented earlier.

**Fig. 6.**
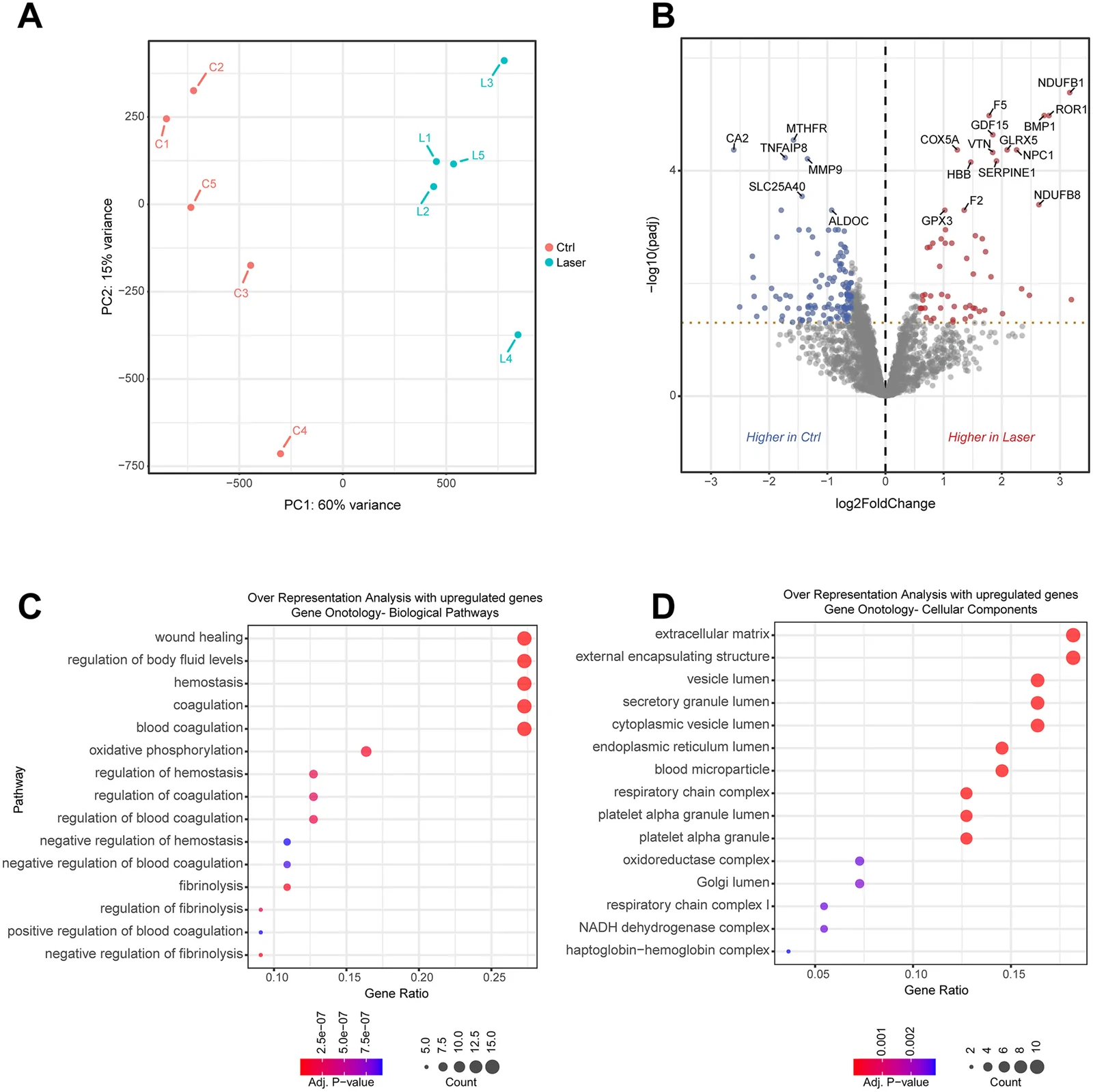
**Proteomics analysis of cardiac spheroids at 7 dpa.** (A) Principal component (PC) analysis demonstrates the separation of control (Ctrl; red) and laser-ablated (Laser; blue) spheroids. (B) Volcano plot of detected proteins with log2 fold change>±0.585 and adjusted *P*-value<0.05, showing the top 20 differential proteins labeled: red, higher in laser; blue, higher in Ctrl. (C,D) Over-representation pathway analysis of proteins using the Gene Ontology Biological Pathways (C) and the Gene Ontology Cellular Components (D) databases, applying a log2 fold change cutoff of 0.585.

### The beating activity of spheroids is affected by laser ablation but recovers over time

We investigated how spontaneous contractile function is affected in response to laser ablation and whether spheroids can recover cardiac contractile features. For this, high-speed movies of groups of spheroids from the same batch were recorded before laser ablation, after laser ablation and at 6 dpa. We used MUSCLEMOTION to analyze the videos and produce plots (Fig. 7A; Movie 1). In parallel, we recorded the same-staged non-lasered spheroids that served as controls. Comparison of the time points before and directly after laser injury revealed, on average, a 4.4-fold decrease in contraction amplitude, a 3.6-fold decrease in contraction duration, a 7-fold decrease in time to peak, a 6.8-fold decrease in relaxation time, a 2.4-fold decrease in peak width at 10% above the baseline, and a 1.3-fold decrease in the parameter of time between peaks. With the exception of the parameter ‘time peak to peak’, the analyzed parameters were significantly different between control and injured spheroids directly after laser injury. However, there were no significant differences in any of the analyzed parameters when comparing laser-ablated spheroids at 6 dpa with the same-stage non-lasered controls (Fig. 7B-G). These results indicate that laser ablation transiently decreased spheroid contractility, but that the effect on contractile function can recover within a period of 6 days.

**Fig. 7.**
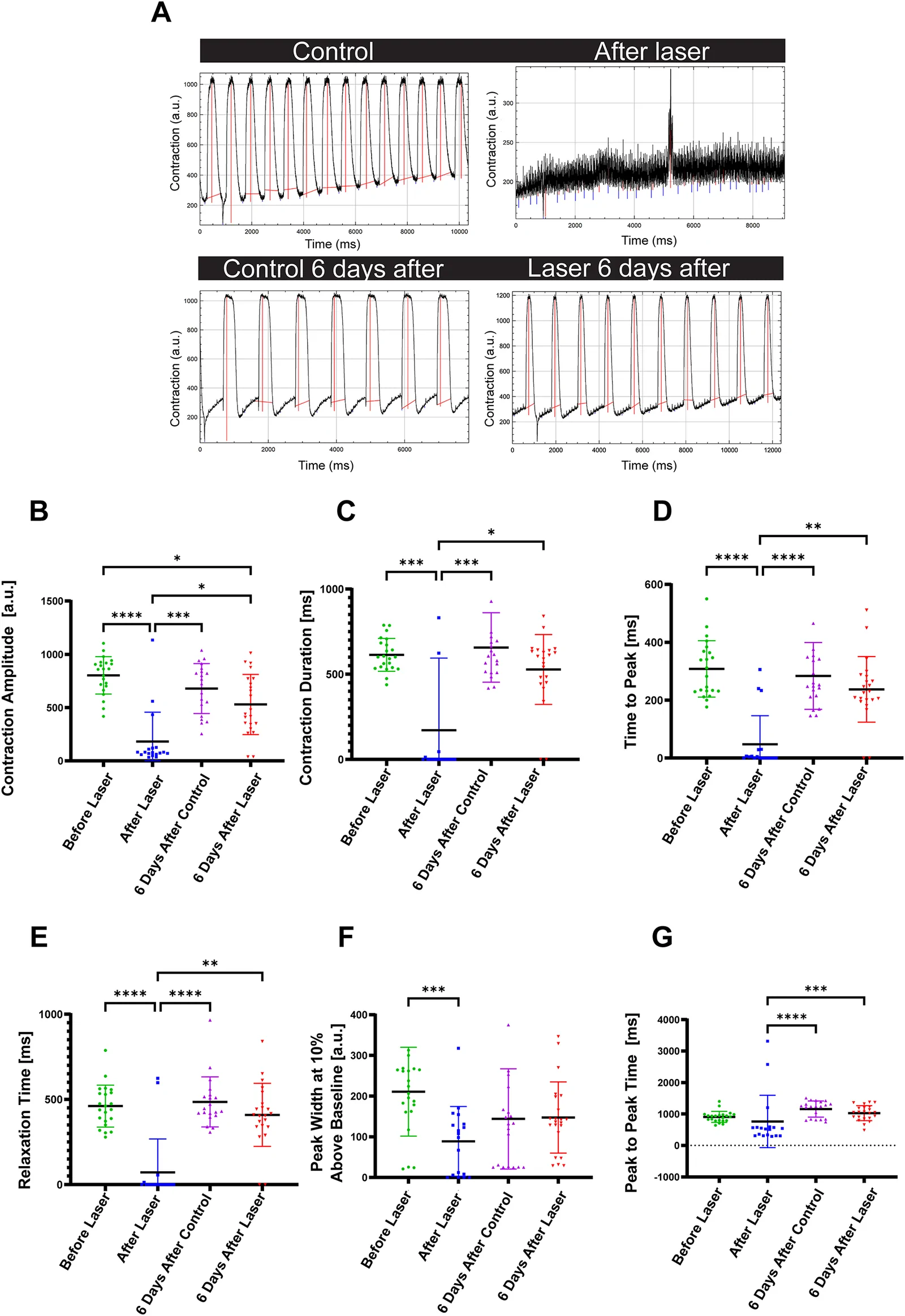
**Assessment of spheroid contractility.** MUSCLEMOTION analysis of beating activity of spheroids before and after being lasered, as well as measurements of both groups 6 days after initial measurements. (A) Examples of contraction plots produced from videos taken at different times before and after laser injury. (B-G) The following parameters were assessed using MUSCLEMOTION: contraction amplitude (B), contraction duration (C), time to peak (D), relaxation time (E), peak width at 10% above baseline (F) and peak to peak time (G) (three biological replicates; before laser *n*=22, after laser *n*=18, 6 days after control *n*=20, 6 days after laser *n*=22). Kruskal–Wallis test. Shown are individual measurements as well as mean±s.d. *P<0.05, **P<0.01, ***P<0.001, ****P<0.0001. a.u., arbitrary units.

## DISCUSSION

In this study, we describe a spheroid-based model of focal cardiac laser injury established from iPSC-CMs and iPSC-CFs. Using this model, we observed localized necrosis and apoptosis after laser injury, mimicking the initial stage of MI. A fibrotic response was observed at the injury site, and contractility was transiently reduced. Injury models established in animals have contributed significantly to the understanding of the mechanisms underlying cardiac injury response and regeneration. However, owing to interspecies differences in cardiac physiology and injury responses, it is essential to investigate these processes within a human-specific context. Interestingly, a limited fibrotic response was confirmed by both immunostaining and proteomics results. Among the top 20 differential proteins enriched in the lasered spheroids are candidates such as SERPINE1, BMP1, VTN, PLG and several coagulation factors, which are generally associated with fibrosis and wound healing and have been described as being upregulated following cardiac ischemia and infarction in patients and in several animal cardiac injury models (Guo et al., 2026; Münch et al., 2017; Yamani et al., 2002; Yao et al., 2026). Other upregulated proteins, such as CCN2 or GDF15, indicate a stress/pro-survival response in the injured microtissues (Ahmed et al., 2011; Dogon et al., 2024). Laser injury was also associated with a shift from glycolytic to oxidative/mitochondrial metabolism in injured spheroids. This finding seems counterintuitive, as embryonic CMs have been shown to strongly rely on glycolysis; however, a recent study has found that oxidative phosphorylation is increased in the injury border zone and is required for CM re-differentiation and successful long-term regeneration in zebrafish (Lekkos et al., 2025). Necrosis- and apoptosis-associated proteins were downregulated at 7 dpa, together with myofibrillar proteins, consistent with the resolution of cell death and loss of CMs at this time point after laser injury.

We propose the use of laser ablation in human cardiac spheroids composed of CMs and fibroblasts as an *in vitro* model that can be used to study reparative and regenerative processes. Photomanipulation is a relatively new technology that is not yet widely used in cardiovascular research compared to, for example, cryo-injury. The latter method is not applicable to sub-millimeter-sized microtissues, as these must be transported in liquid, and cryo-conditions would immediately freeze the entire tissue. Photomanipulation using a laser through a microscope is applicable at the cellular level and can be performed semi-automatically, thereby reducing operator-dependent variability. The same region exposed to UV laser light can be used in each well of a 96-well plate, enabling mass treatment and screening of microtissues. Our study in a scaffold-free spheroid model system differs in a number of technical (laser wavelength and exposure modality), analytical (machine learning-assisted semi-automatic image analysis) and biological (size, fibroblast origin, ECM composition) features from those previously published. Although adding additional cell types, such as endothelial cells, might mimic the original tissue's composition better, the role of the endothelial component is not very well defined in spheroid models: endothelial cells do not form complete vascular systems, and oxygen supply by diffusion is sufficient in these small microtissues (Hirschhaeuser et al., 2010). Therefore, we decided to include two cell types, iPSC-CMs and iPSC-CFs. The addition of iPSC-CFs allows study of the consequences of a cardiac injury on the ECM. Although we used iPSC-CFs here, it would be possible to modify the spheroid assembly using patient-derived fibroblasts to interrogate personalized profibrotic responses (Błyszczuk et al., 2020). Indeed, the observation that focal laser injury resulted in a relatively weak fibrotic response may be due to the developmental age of the fibroblasts, as suggested by a study comparing the properties of cardiac microtissues containing primary fetal or adult murine cardiac fibroblasts (Li et al., 2017). The addition of immune cells is another interesting option, which has recently been shown to play an important role in ECM deposition and remodeling in cardioids undergoing cryo-injury (Ceci Ginistrelli et al., 2026 preprint). The herein presented iPSC-CM and iPSC-CF spheroids represent a simple model that allows genetic modification of specific cell types and study of cellular interactions during injury response. Some parameters, such as spheroid size, contractile activity and EGFP fluorescence, can be measured in a straightforward and non-invasive way during culture and treatments. Therefore, the system is amenable to toxicological applications, small-molecule screenings and the study of pro-regenerative compounds.

We did not observe an increase in cell proliferation, nor a full recovery of spheroid size or EGFP fluorescence from the CM reporter in response to laser injury, suggesting a lack of intrinsic capacity for cardiac spheroids to re-enter the cell cycle. This finding was also confirmed by proteomics analysis, which showed no markers clearly associated with cell cycle progression in the dataset. However, motion analysis demonstrated the recovery of contractile activity in iPSC-CMs. Taken together, these findings suggest that although spheroids lacked regenerative capacity, functional recovery could still occur. Although the exact mechanism remains unclear, it can be speculated that functional recovery occurs independently of complete tissue regeneration. The remaining viable tissue appears capable of recovering from injury within 6 days following laser ablation. The lack of intrinsic capacity for proliferation makes this an attractive model to test the pro-regenerative capacity of chemical compounds or genes. Given the production speed and small dimension, it represents a model for scalable screening assays for cardiac regenerative research.

## MATERIALS AND METHODS

### iPSC lines and their maintenance

The iPSC line WTC-mEGFP-TNNI1-cl172 (Allen Institute, distributed by Coriell Institute for Medical Research, Camden, NJ, USA) (Roberts et al., 2017), referred to as TNNI1-EGFP, was used to generate iPSC-CMs. The iPSC line GM25256 (Coriell Institute for Medical Research), originating from the same apparently healthy adult male donor (Kreitzer et al., 2013), was used to generate iPSC-CMs without EGFP reporter expression and iPSC-CFs. The latter iPSC line is referred to as WT throughout the paper. Quality and safety testing of used iPSC cell lines was confirmed by the supplier. iPSCs were cultured in complete mTeSR1 medium (STEMCELL Technologies, 85850) supplemented with Antibiotic-Antimycotic (Thermo Fisher Scientific, 15240096) in a humidified incubator at 37°C with 5% CO_2_. Culture plates (Corning/Falcon, 353046) were coated with human embryonic stem cell-qualified Matrigel (Corning, 354277). Upon reaching confluency, cells were passaged as clumps using ReLeSR (STEMCELL Technologies, 100-0484). The cells were cryopreserved with mFreSR™ (Stem Cell Technologies, 5854).

### Differentiation protocol for iPSC-CMs

iPSC-CMs were differentiated according to Lian et al. (2013) with modifications. Briefly, once iPSCs reached 70-80% confluency, the medium was switched from mTeSR1 (Stem Cell Technologies, 85850) to RPMI1640 (Gibco, 11875119) with B27 supplement minus insulin [Gibco, A1895601, RPMI/B27(−)] and 7.5 μM CHIR99021 (Selleckchem, S2924) (day 0). On day 2, the medium was replaced with RPMI/B27(−) containing 7.5 μM IWP-2 (Sigma-Aldrich, CAS 686770-61-6). After 48 h, cells were maintained in RPMI/B27(−) without additives for 2 days. From day 6 to day 15, the medium was refreshed every other day with RPMI 1640 containing serum-free B27 [RPMI/B27(+)]. CM enrichment was performed using glucose-free RPMI 1640 (Biological Industries, 01-101-1A/BI) supplemented with B27(+) and sodium DL-lactate solution (SCBT, sc-301818A plus HEPES, Sigma-Aldrich, H0887) for 7 days. Following a 2-day recovery in RPMI/B27(+), iPSC-CMs were either cryopreserved using STEMdiff™ CM Freezing Medium (STEMCELL Technologies, 5030) or directly used for experiments.

### Differentiation protocol for iPSC-CFs

A protocol based on that of Zhang et al. (2019), with some modifications to iPSC culturing, was used. The medium for iPSCs at 70-80% confluency (within 5 days post-plating) was changed from mTeSR1 to RPMI/B27(−) with 6 μM CHIR99021 (day 0). After 24 h, the medium was replaced with RPMI/B27(−) without additives, followed by RPMI/B27(−) with 5 μM IWR-1 (Selleckchem, S7086). After 48 h, cells were cultured in RPMI/B27(−) without supplements. From day 6 onward, the six-well plates were coated with Matrigel^®^ Basement Membrane Matrix (Corning, 354234).

On day 6, cells were detached and replated at 20,000 cells/cm^2^ in a six-well plate with Advanced DMEM (Gibco, 12634-028)/GlutaMAX (Gibco, 35050-061) containing 5 μM CHIR99021, 2 μM retinoic acid (Sigma-Aldrich, R2625), 5 μM Y27632 (Selleckchem, s1049) and 1% fetal bovine serum (FBS; Gibco, 26140079). On day 7, the medium was replaced with Advanced DMEM/GlutaMAX supplemented with 5 μM CHIR99021 and 2 μM retinoic acid. After 48 h, the cells were treated with Advanced DMEM/GlutaMAX without additives. On day 11, cells were detached and passaged at a 1:4 ratio in Advanced DMEM/GlutaMAX with 2 μM SB431542 (Tocris, 1614). From day 12 to day 15, the medium was refreshed every other day with advanced DMEM/GlutaMAX containing 2 μM SB431542. When reaching confluency (typically on day 15), cells were replated at 10,000 cells/cm^2^ in Fibroblast Growth Medium 3 (PromoCell, C23025; FGM3) supplemented with 20 ng/ml FGF2 (R&D Systems, 233-FB) and 10 μM SB431542. After 48 h, the medium was changed. On day 19, cells were again passaged at a density of 10,000 cells/cm^2^ in FGM3 with 20 ng/ml FGF2 and 10 μM SB431542. From day 21 onward, the medium was changed every other day with FGM3 containing 10 μM SB431542. After reaching confluency, cells were either cryopreserved or used for further experiments.

### Assembly of cardiac spheroids

Cardiac spheroids were generated by adding a total of 5000 cells per well to BIOFLOAT™ 96-well plates (faCellitate, F202003) at a ratio of 75% iPSC-CMs and 25% iPSC-CFs. Culture medium consisted of CM plating medium made with RPMI1640, B27(+), knockout serum replacement (Thermo Fisher Scientific, 10828028), thiazovivin (Sigma-Aldrich, SML1045) and antibiotics (Gibco, 15240062), mixed 50:50 with FGM3 (PromoCell) for the seeding of spheroids and the next 48 h, and of CM maintenance medium consisting of RPMI1640, B27(+) and antibiotics mixed 50:50 with FGM3 for the following medium changes. Additionally, a medium based on high-glucose DMEM supplemented with 10% FBS (Gibco, 26140079) was used for another set of experiments, each performed in triplicate. This medium did not contain FGF2 and included a twofold increase in L-ascorbic acid 2-phosphate (AA2P; 28 μM) and thus should predispose to fibrosis (Hata and Senoo, 1989). For the assessment of the pro-fibrotic effect of TGFβ, we used the same medium supplemented with 10 ng/ml TGFβ, with medium exchange every 2 days (Fig. S3). Spheroids were cultured for 7 days before laser treatment; see Fig. 1A for the timeline of the experiments.

### Laser ablation of cardiac spheroids

Before imaging and laser ablation, the spheroid medium was changed to Phenol Red-free and serum-free DMEM (Gibco, 31053028) and then returned to the original spheroid medium after laser ablation. The 96-well plate containing spheroids was placed in an Acquifer Imaging Machine (Bruker), and the internal temperature of the device was set to 37°C. After centering the spheroid positions in each well using images obtained with a Nikon CFI Plan-Achromat UW 2× lens, GFP and bright-field images were captured, and laser ablation was performed using a Nikon CFI Super Plan Fluor 20×/0.45 NA WD 8.2-6.9 lens. The Acquifer Imaging Machine was equipped with a photomodulation module with a 405 nm continuous-wave laser diode (Rapp OptoElectronic, Wedel, Germany). According to the manufacturer, when set at 100%, a power reading of 26.5 mW at a beam spot size of ∼3 μm diameter was measured at the objective plane of the 20× objective using a Thorlabs S140C handheld optical power meter, resulting in a calculated power density of 3.75×10^5^ W/cm^2^. For laser ablation, a uniform polygonal region of 15,000 μm^2^ was defined for all spheroids in this study, and the position was checked for each well. Laser ablation was performed by scanning over the chosen laser area using the following settings in the software interface: step size 10, two iterations, maximum intensity. Post-ablation, 20× GFP and bright-field images were reacquired under the same conditions as before ablation.

### Immunofluorescence staining of cryosections and 2D-cultured cells

2D-cultured cells were immunostained as previously reported (Beauchamp et al., 2020). For immunofluorescence microscopy of cryosections, spheroids were washed with PBS and fixed in 3% paraformaldehyde for 30 min. After overnight incubation in 30% sucrose in PBS at 4°C, spheroids were embedded in OCT compound (Leica Biosystems, Richmond, IL, USA), and sections were obtained using a Leica CM1950 cryostat set to a thickness of 8 μm. Cryosections on slides were incubated with 0.5% Triton X-100 in PBS for 10 min, then washed and blocked using blocking buffer consisting of 2% bovine serum albumin (BSA; Sigma-Aldrich, A647) in PBS with 1% Nonidet-P40 for 1 h at room temperature and then incubated overnight at 4°C with primary antibodies in blocking buffer. For primary and secondary antibodies, see Table S11. DAPI for counterstaining nuclei was added to the secondary antibody solution (NucBlue, Thermo Fisher Scientific, R37606). All secondary antibodies were diluted 1:1000 in blocking buffer and incubated with cryosections for 1 h at room temperature in the dark. Preparations were then washed with PBS and embedded using ibidi Mounting medium with DAPI (ibidi, 50011). Preparations were examined using either a Leica TCS SP8 confocal microscope with a PL APO 20×/0.75 NA water lens, or a Zeiss LSM710 confocal microscope with an EC Plan-Neofluar 40×/1.30 NA oil immersion lens for Fig. S2.

### Immunofluorescence and confocal microscopy of cleared spheroids

For the clearing of fixed spheroids, the protocol ‘ScaleS’, according to Nürnberg et al. (2020) was used with modifications. Briefly, fixed spheroids were incubated with D-sorbitol, glycerol and DMSO in PBS at 37°C, followed by several changes of solutions containing urea (4 M and 8 M urea, glycerol, Triton X-100 in ddH_2_O). Spheroids were first incubated with primary antibodies, washed and then incubated with secondary antibodies in blocking solution (2.5% BSA, 0.05% Tween-20, 0.1% Triton-X-100 in PBS with 0.2% sodium azide) for 3 days. Imaging was performed with spheroids in a 15-well slide with glass bottom (ibidi, 81507) in a solution of 40% D-sorbitol, 10% glycerol, 4 M urea and 15% DMSO. Preparations were examined using a Leica TCS SP8 confocal microscope with a PL APO 20×/0.75 NA water lens. Approximately 60-100 optical sections at steps of 2 μm were recorded and further analyzed. Two examples of image stacks obtained from cleared spheroids with and without laser injury are shown in Movies 2 and 3. Orthogonal side views of image stacks and maximum intensity projections were generated in Leica LASX software. Contrast enhancement was applied to the figures shown, but quantification was always performed using raw images. To corroborate the immunofluorescence staining observed in whole-mount preparations, we also performed immunofluorescence staining using cryosections of spheroids 3 and 7 days after laser ablation (Fig. 3).

### Cell viability and cell death assays

A LIVE/DEAD™ Viability/Cytotoxicity Kit (Invitrogen, L3224) was used for the assessment of necrosis in live spheroids. EthD-1 indicates necrotic cells, and calcein-AM indicates metabolically active cells. For this experiment, WT iPSC-CMs without the expression of a GFP reporter protein were used. Following laser ablation, the spheroids were washed with 1× PBS. Then, 4 μM EthD-1 in PBS and 2 μM calcein-AM in PBS were added to each well, and the spheroids were incubated for 40 min at room temperature in the dark. After incubation with dyes, the spheroids were transferred to a glass-bottom dish and imaged using a Leica TCS SP8 confocal microscope with a PL APO 20×/0.75 NA water lens. Spheroids are shown as a maximum-intensity projection of 44 optical sections produced in Leica LAS-X software. For the TUNEL assay, an *in situ* cell death detection kit using tetramethylrhodamine (TMR) Red (Roche, Merck, 12156792910) was used on cryosections obtained from spheroids 3 days after injury. As a positive control, DNase (Merck, 260913) treatment was used. Cryosections were mounted on slides and embedded using ibidi mounting medium with DAPI (ibidi, 50011). A Nikon Eclipse Ti-E wide-field microscope was used to capture images of the sections. For the detection of caspase-3/7 activity, the spheroids were stained with CellEvent™ Caspase-3/7 Detection Reagent (Thermo Fisher Scientific, C10423) right after the laser injury. Live-stained spheroids were analyzed using a Nikon inverted microscope with a 20× lens and the FITC filter set. 10 μl CellEvent™ caspase (2 mM) in DMSO was diluted in 990 μl Phenol Red-free DMEM (100× dilution), then incubated with spheroids for 1 h at 37°C. A Nikon Eclipse Ti-E widefield microscope was used to take pictures of the live spheroids. The fluorescent images from TUNEL and Caspase-3/7 activity assay were analyzed in ImageJ. Following the removal of the background, the area of spheroids was manually outlined, and the integrated density was measured. Normalized values were used for statistical analysis in GraphPad Prism.

### Video analysis of spheroid contractions

Video analysis was performed as previously published (Beauchamp et al., 2020). Briefly, a modified GoPro Hero Black 6 camera (Back-Bone Gear Inc., Kanata, Canada) was used to record short video sequences at a high frame rate of 240 frames/s. Spheroids were transferred to a heating chamber with a warmed lid and temperature controller (ibidi, IBD-10918) on the stage of an inverted microscope (Nikon Eclipse TS100) with a Nikon 10× Ph1 ADL/1.2 phase-contrast lens and were allowed to warm up to 37°C for 10 min before recording. The open-source macro MUSCLEMOTION for ImageJ was then used to extract motion data (van Meer et al., 2018). Beating activity was assessed immediately before, directly after and 6 days post-laser ablation (Fig. 7).

### GFP intensity and size analysis of 2D spheroid images

GFP images of the spheroids were acquired on a Bruker Acquifer Imaging system at 20× magnification with a resolution of 2048×2048 pixels and a 16-bit grayscale depth, using the same exposure time and illumination settings for each session. Imaging was performed immediately before laser ablation, immediately after laser ablation, and 3 days or 7 days after laser ablation.

Individual Acquifer images were segmented with Convpaint (Hinderling et al., 2024) using a custom Python script for batch processing. Spheroids were labeled as foreground, and a DINOv2+Gaussian feature extractor was trained on sparse annotations from a set of five Acquifer images. Segmentation performance was quantitatively assessed by comparing model-generated masks to independent manual annotations of the high-resolution spheroid images: ground-truth segmentations for nine randomly selected spheroids were manually delineated, and the agreement between predicted and reference masks was evaluated using a balanced F-score (F1 score; Fig. S4A). A custom Python script was used to parse Acquifer filenames and to merge well images and corresponding spheroid segmentations separately along the time axis. Time-resolved individual spheroid sizes and intensity statistics were then extracted using the extract regionprops to CSV processing function in the napari-tmidas batch image processing pipeline (Meer, 2025).

For the GFP intensity and size analysis of 2D spheroid images, a Mann–Whitney analysis was performed to compare control and laser groups for each time point independently. The Wilcoxon matched pairs signed rank test was employed to compare measurements within the control group across different time points and, likewise, within the laser group. Values were normalized to the mean baseline value of the control and laser groups (three biological replicates; for 3 days with overall 143 spheroids and for 7 days with overall 139 spheroids). The results of the statistical analysis are reported in Table S1. Power analysis was conducted in R (4.4.0) using the pwr (1.3.0; Champely, 2006) and lsr (0.5.1; Navarro, 2015) packages to estimate effect sizes (Cohen's *d*), achieved power and required sample sizes for group comparisons. For each time point, the laser and control groups were compared with two-sample *t*-tests; within each treatment group, all pairs of time points were also compared using two-sample *t*-tests. Bonferroni-adjusted significance levels were used to account for multiple testing, and all calculations were based on observed group means and standard deviations. The results of the power analysis are reported in Tables S2 and S3.

### Ki67 counting and fibrosis marker intensity analysis of 3D spheroid images

Leica image files (LIF) were converted to the TIF format using the napari-tmidas (Meer, 2025) batch microscopy image conversion pipeline. Color channels were then separated using the split color channels processing function in the napari-tmidas batch image processing pipeline. GFP images of 3D spheroids were cropped and segmented via the napari-tmidas crop anything pipeline, which leverages Segment Anything 2 (Ravi et al., 2024). Spheroid masks were refined to half-spheroids using the keep slice range by area processing function in the napari-tmidas batch image processing pipeline. These half-spheroid masks were subsequently partitioned into three layers of equal thickness (inner, middle, outer) and sum (Fig. S4B) using the subdivide labels into layers processing function in the batch image processing pipeline. Sum hereby indicates the unpartitioned half spheroid volume. The DAPI counterstaining could not be used for nuclei segmentation owing to inhomogeneous staining.

Fibrosis marker intensity statistics and layer sizes of each half-spheroid were quantified using the batch ROI colocalization pipeline in napari-tmidas (Fig. S4B). Ki67-channel images were segmented with Convpaint using a custom Python script for batch processing. For this, voxels were classified against a three-class scheme: spheroid foreground, Ki67-positive signal and image background. A Dinov2+Gaussian feature extractor was then trained on sparse three-class annotations from a set of five spheroid images. The trained model was then used in a custom Python notebook to batch process all spheroid images and to produce instance segmentations in which each Ki67-classified blob was given a unique label ID (Fig. S4B). Blobs containing weak Ki67 intensity were filtered out using the filter labels by intensity processing function in the napari-tmidas batch image processing pipeline, which employs k-medoids clustering. For each spheroid, the filter threshold was set as the midpoint between the lowest and second-lowest medoids from a three-medoids clustering. Ki67-positive blobs were then counted in each of the three spheroid layers, and their intensity statistics were derived using the batch ROI colocalization pipeline in napari-tmidas.

### Proteomics

Control and lasered spheroids were collected at 7 dpa, snap frozen and processed for proteomic analysis (*n*=24, five biological replicates). Samples were analyzed by data-independent acquisition (DIA) liquid chromatography–tandem mass spectrometry at the Proteomics and Mass Spectrometry Core Facility (PMSCF), University of Bern. Raw DIA data were processed in Spectronaut (Biognosys, Schlieren, Switzerland) (hybrid directDIA+‘Deep’ search mode). Peptides were assembled into protein groups using a parsimony principle, with a controlled protein group false discovery rate of 1% (precursor and protein *q*-values and posterior error probabilities all set to 0.01). A leading protein/gene was assigned to each protein group as the member with the best peptide coverage. Common contaminants (keratins, trypsin, etc.) included in the search database were flagged and removed from downstream analysis. Only protein groups identified by ≥2 peptides and detected in at least the minimum number of replicates in at least one group (minNumReps=3 of 5), were retained for statistical testing (4080 protein groups).

Abundance metrics were computed per protein group based on ion-quantification-based MaxLFQ-type abundance (IQ), calculated with the R package ‘iq’ (v2.0.1; Pham et al., 2020) after median renormalization of the filtered fragment set. Missing values were imputed protein-wise: for proteins with ≤3 non-zero values in a group, remaining missing values were imputed by a left-censored method (Gaussian draw, width 0.3× sample s.d., centered at sample mean−2.5× sample s.d.), with any remaining missing values imputed by maximum likelihood estimation. Differential expression between laser and control groups (unpaired, contrast ‘Laser−Ctrl’) was tested on the imputed IQ (iIQ) matrix using a moderated empirical Bayes test [limma (v3.66.0; Ritchie et al., 2015), R v4.5.2]; significance was defined as Benjamini–Hochberg adjusted *P*<0.05. Pathway analysis was performed using overrepresentation analysis (ORA) clusterProfiler (v4.18.4; Yu et al., 2012) against Gene Ontology (GO) and Reactome pathway databases (Ashburner et al., 2000; Ragueneau et al., 2026; The Gene Ontology Consortium, 2026). For the laser versus control comparison, proteins with adjusted *P*-value<0.05 and |log2 fold change|>0.585 were tested by ORA. The full set of results is shown in Tables S6-S10.

### Statistical analysis

Statistical analyses were performed using Prism 10 (GraphPad Software) and R (R Core Team, 2026). Data were assessed for normality and lognormality, and non-parametric tests (Kruskal–Wallis, Mann–Whitney or Wilcoxon matched-pairs signed rank tests) were applied when distributions deviated from normality (Table S1). The statistical test used for each dataset is specified in the respective figure legends. Mixed model analyses for expression analysis depicted in Fig. 4B,C, Fig. 5B-G and Fig. S3A-E were performed in R (version 4.4.0). Statistical models for each measurement were selected using an evidence-based model-selection procedure. For each response variable, three candidate mixed-effects models were considered: a Gaussian linear mixed model (LMM), a Gamma generalized linear mixed model (GLMM; log link) and a Log-normal LMM (fit to log-transformed values). The latter two were considered only when the response was strictly positive. All structurally feasible candidates were fitted independently rather than sequentially [lme4 (1.1.38; Bates et al., 2015), lmerTest (3.2.0; Kuznetsova et al., 2017)]. Each candidate was evaluated on (1) raw-data characteristics [skewness, kurtosis and the Akaike weight of a structure-free distributional fit; fitdistrplus (1.2.1; Delignette-Muller and Dutang, 2015)], (2) explanatory power [marginal and conditional *R*^2^, Nakagawa's method; performance (0.16.0; Lüdecke et al., 2021)], (3) residual diagnostics [Shapiro–Wilk test for the Gaussian model; simulated-residual tests for uniformity, dispersion, and outliers for the Gamma and Log-normal models; DHARMa (0.5.0; Hartig, 2026)] and (4) fit stability (optimizer/convergence warnings). Each criterion was scaled to a common evidence contribution and combined into a single weighted evidence score per candidate; the candidate with the strongest total evidence was retained for inference. Models were excluded only for structural reasons – namely, failure to converge or a singular fit, and never based on residual diagnostics alone. For any candidate for which primary residual diagnostic indicated a poor fit, a random-slopes variant of the same distributional family was additionally fitted and included in the comparison if it converged without singularity. Fixed effects in the selected model were tested using Type III tests [Satterthwaite-approximated F-tests for Gaussian/Log-normal models; Wald χ^2^ tests for Gamma models; car (3.1.5; Fox and Weisberg, 2018)]. Post-hoc pairwise comparisons were performed with emmeans (2.0.3; Lenth et al., 2026) (either ‘for all pairwise combinations of treatment levels’ for treated spheroids or ‘for a pre-specified set of comparisons’ for layered analysis), with *P*-values adjusted for multiple comparisons (Bates et al., 2015). Data handling used dplyr (1.2.0); figures were generated with ggplot2 (4.0.2; Wickham, 2016) and patchwork (1.3.2; Pedersen, 2025). Evidence scores were calculated as a weighted sum of nine criteria, each scaled to a common 0-1 range (higher values indicating stronger support for a given candidate): positivity of the response, right-skewness and kurtosis relative to a normal distribution (weights 2, 2 and 1, respectively); the Akaike weight of the structure-free distributional fit (weight 1); marginal *R*^2^, conditional *R*^2^ and the intraclass correlation coefficient (weights 2, 1 and 1, respectively); residual diagnostics (weight 3); and fit stability (weight 2). Weights were fixed *a priori* and applied identically across all measurements and candidates. Where a given criterion could not be computed for a candidate, its weight was redistributed proportionally among the remaining available criteria so that the total weighted score remained comparable across candidates. The results of the statistical analysis are reported in Tables S4 and S5.

### Use of AI tools

ChatGPT, Claude, Perplexity and Gemini were used for coding troubleshooting related to statistics, spelllchecking and proteomic analysis. All such generated outputs were manually reviewed and edited as needed, and the authors take responsibility for the contents of the article.

## Supplementary Material

10.1242/dmm.052855_sup1 Supplementary information

Table S6. Differentially expressed proteins between control and lasered spheroids.

Table S7. Overrepresentation analysis (ORA) with upregulated proteins using Gene Ontology Biological Pathways.

Table S8. Overrepresentation analysis (ORA) with downregulated proteins using Gene Ontology Biological Pathways.

Table S9. Overrepresentation analysis (ORA) with upregulated proteins using Gene Ontology Cellular Components.

Table S10. Overrepresentation analysis (ORA) with downregulated proteins using Gene Ontology Cellular Components.
