# Supplementary material for: Development of a laser injury model using human cardiac spheroids
Source: Dis Model Mech. 2026 Oct 28;19(10):dmm052855. doi: 10.1242/dmm.052855 (PMC13750404; doi:10.1242/dmm.052855)
Supplement: Supplementary file 1 [file dmm-19-052855-s1.pdf]

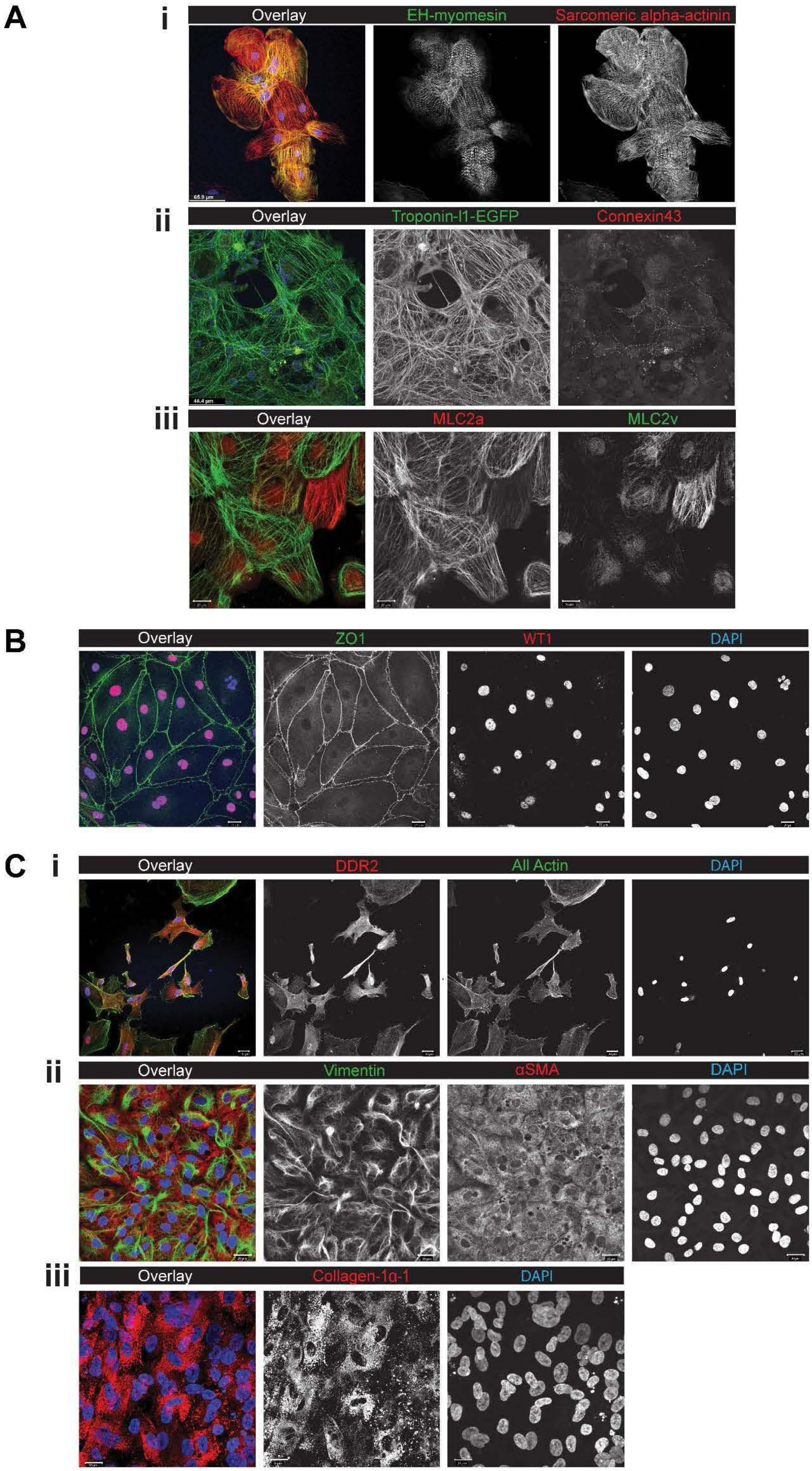

Disease Models & Mechanisms • Supplementary information

Fig. S1. Characterization of iPSC-derived cells by immunocytochemistry. (A) Confocal microscope images of iPSC-CMs immunostained for EH-myomesin (green), sarcomeric α-actinin (red) (i) Troponin-I1-EGFP (green), connexin43 (red) (ii) and myosin light chain 2a (MLC2a) (green), myosin light chain 2v (MLC2v) (red) (iii). (B) Confocal microscope images of iPSC-derived epicardial progenitor cells (EPCs) stained with ZO-1 (green), WT-1 (red). (C) Confocal microscope images of iPSC-CFs immunostained for DDR2 (red), all actin (green) (i), vimentin (green), αSMA (red) (ii), collagen-1α-1 (red) (iii), and their overlay images, respectively. In all panels, the blue channel shows nuclear counterstaining with DAPI, scale bars, 20 µm.

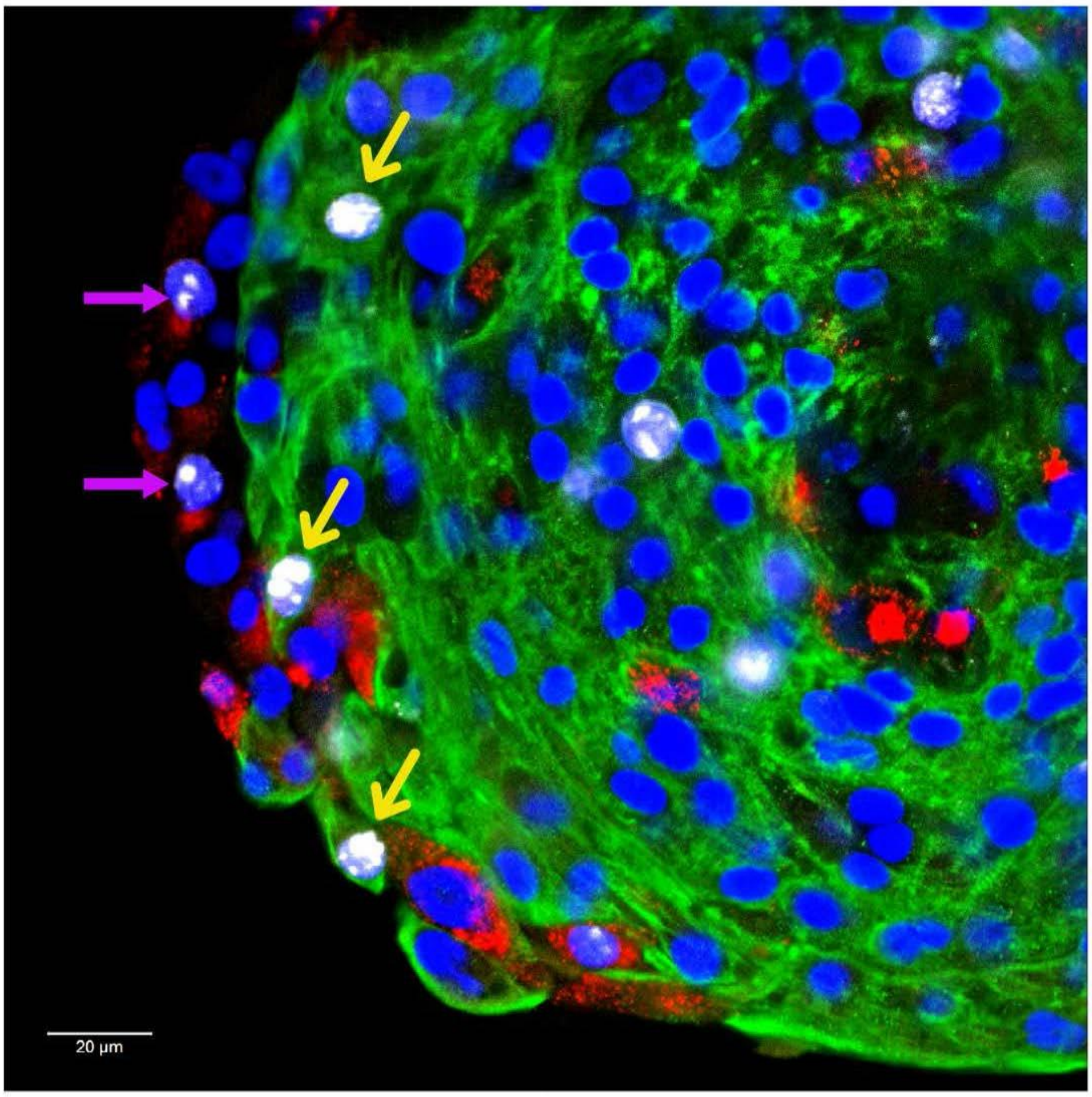

Fig. S2. Baseline proliferation markers in different cell types. Shown is a single optical confocal microscope section (using a 40x/1.30 oil lens) recorded from an uninjured, cleared cardiac spheroid immunostained for troponin-I1-EGFP (green) as a marker of iPSC-CMs, for collagen I α 1 (red) as a marker for iPSC-CF, and for Ki67 (white) as a marker for proliferative cells. The blue channel shows counterstaining with DAPI. Yellow arrows point to cells showing Ki67-positive iPSC-CMs, arrows in magenta point to cells showing colocalization of Ki67-positive iPSC-CFs. Scale bar, 20 µm.

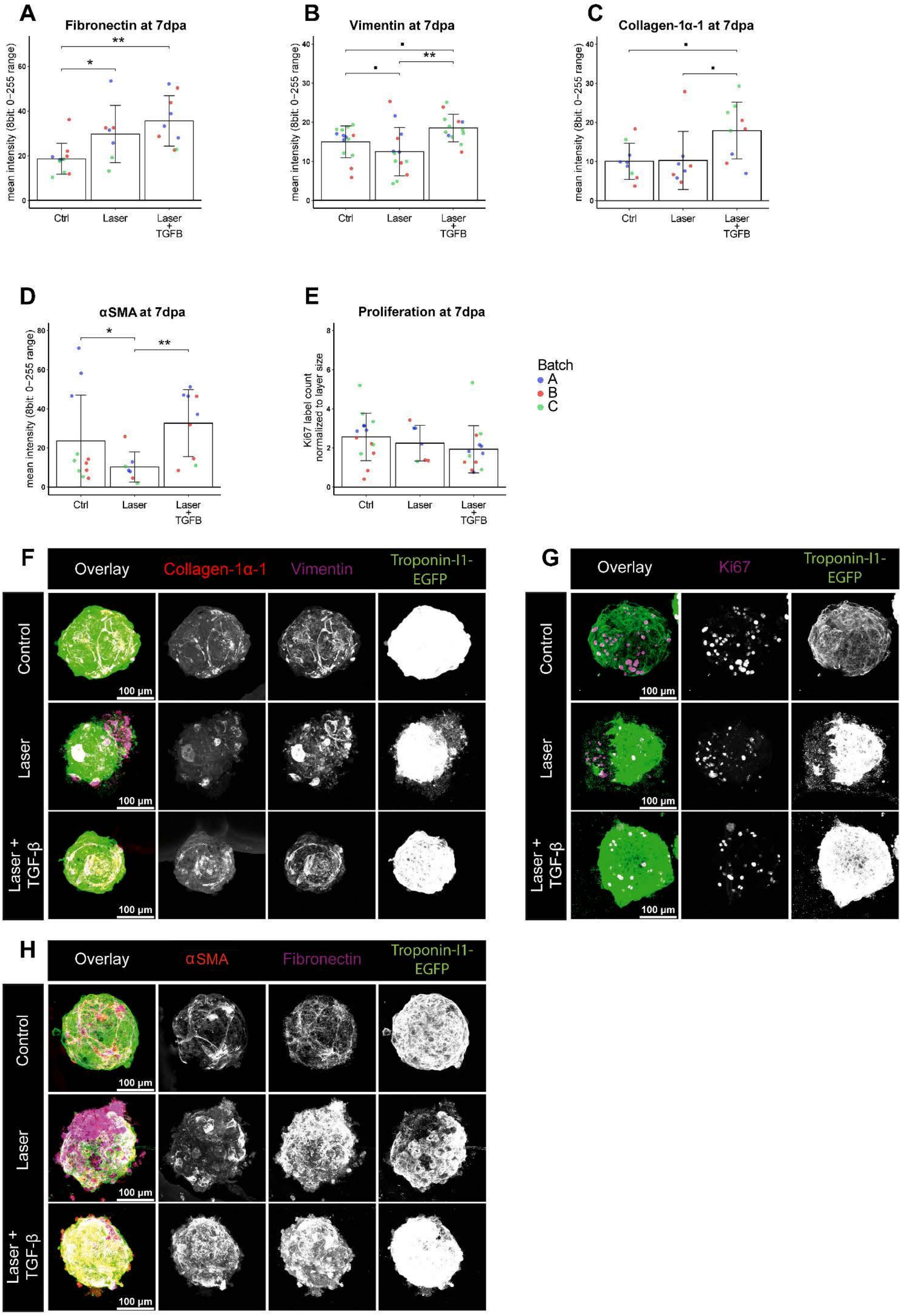

Disease Models & Mechanisms • Supplementary information

Fig. S3. Effect of TGF-β treatment on laser-induced injury response. Spheroids were cultured in high glucose DMEM supplemented with 10% FBS and 28 µM AA2P. At 7 days post-ablation (dpa), spheroids were fixed and used for immunostaining against fibrotic markers and to detect cell proliferation. (A-D) Quantitative analysis of fibrosis marker mean intensity (8-bit: 0-255) within three treatment conditions (Ctrl= Control, Laser= laser injured, TGF-β = laser injured + TGF-β treated (10 ng/ml)). Each data point refers to one spheroid. Shown is the mean marker intensity within one half of the spheroid volume. Shown are quantifications of fibrotic markers for fibronectin (A), vimentin (B), collagen-1α-1 (C), and αSMA (D). (E) Quantitative analysis of proliferation assessed by Ki67 label counts. Overall, 7 to 14 spheroids were used per treatment. Shown are individual measurements as well as mean ± SD, **$P < 0.01$, *$P < 0.05$, ˙$P < 0.1$. Significance was assessed via mixed-effects models as described in the Methods section under statistical analysis. Blue, red, and green dots represent samples from different spheroid aggregation batches, which are considered biological replicates. (F-H) Representative maximum intensity projections of confocal stacks shown as overlay and separated image channels in greyscale as used for the quantifications (3 biological replicates). Spheroids were stained for collagen-1α-1 (red), vimentin (magenta) and troponin-I1-EGFP (green) (F), Ki67 (magenta) and troponin-I1-EGFP (green) (G) and αSMA actin (red), fibronectin (magenta) and troponin-I1-EGFP (green) (H). Scale bars, 100 µm.

Disease Models & Mechanisms • Supplementary information

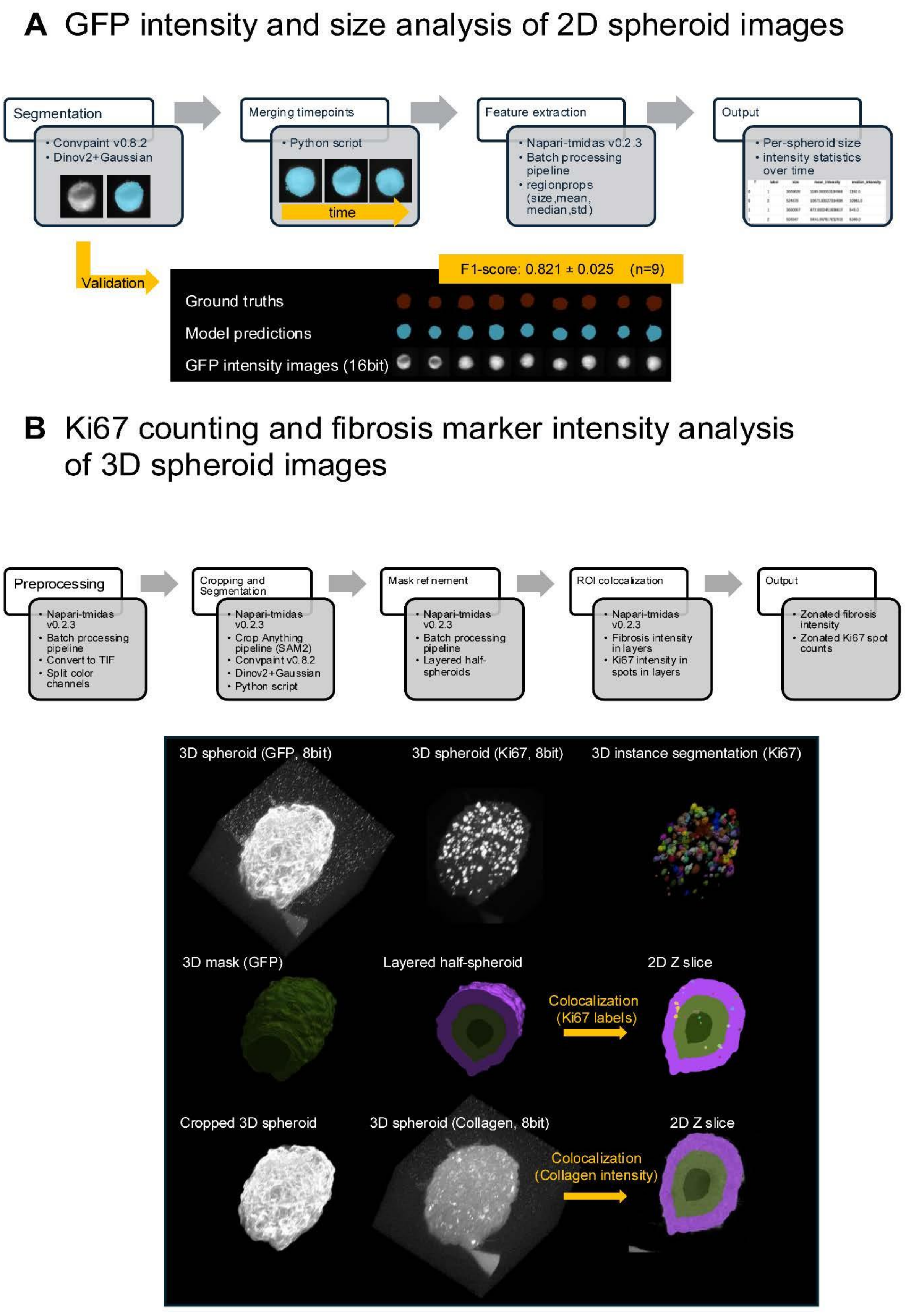

Disease Models & Mechanisms • Supplementary information

Fig. S4. Spheroid image analysis pipelines. (A) Batch processing of 2D spheroid images acquired with the Acquifer Imaging Machine. Segmentation was performed using Convpaint, while all subsequent processing steps and segmentation validation were executed through napari-tmidas pipelines. (B) Batch processing of 3D spheroid images acquired with a Leica TCS SP8 confocal microscope. All processing steps were performed using napari-tmidas pipelines.

Table S1. Biostatistical analysis of size and GFP signal assessment in spheroids.

Graph	Compared groups	Test	P value	P value summary	Significant
Size 3 days	Laser before/Control before	Mann-Whitney	0.94	ns	No
Size 3 days	Laser 3 days after/Control 3 days after	Mann-Whitney	<0.0001	****	Yes
Size 3 days	Laser after/Control after	Mann-Whitney	0.435	ns	No
Size 3 days	Control after/Control 3 days after	Wilcoxon matched-pairs signed rank	<0.0001	****	Yes
Size 3 days	Control before/Control 3 days after	Wilcoxon matched-pairs signed rank	0.7302	ns	No
Size 3 days	Laser before/ Laser after	Wilcoxon matched-pairs signed rank	<0.0001	****	Yes
Size 3 days	Laser 3 days after/Laser after	Wilcoxon matched-pairs signed rank	<0.0001	****	Yes
Size 3 days	Laser 3 days after/Laser before	Wilcoxon matched-pairs signed rank	<0.0001	****	Yes
Size 3 days	Control after/ Control before	Wilcoxon matched-pairs signed rank	<0.0001	****	Yes
Size 7 days	Laser before/Control before	Mann-Whitney	0.9715	ns	No
Size 7 days	Laser 7 days after/Control 7 days after	Mann-Whitney	<0.0001	****	Yes
Size 7 days	Laser after/Control after	Mann-Whitney	0.5835	ns	No
Size 7 days	Laser before/ Laser after	Wilcoxon matched-pairs signed rank	0.0076	**	Yes
Size 7 days	Laser 7 days after/Laser before	Wilcoxon matched-pairs signed rank	<0.0001	****	Yes
Size 7 days	Laser 7 days after/Laser after	Wilcoxon matched-pairs signed rank	<0.0001	****	Yes
Size 7 days	Control before/Control 7 days after	Wilcoxon matched-pairs signed rank	0.1395	ns	No
Size 7 days	Control after/Control 7 days after	Wilcoxon matched-pairs signed rank	<0.0001	****	Yes
Size 7 days	Control after/ Control before	Wilcoxon matched-pairs signed rank	<0.0001	****	Yes

Disease Models & Mechanisms • Supplementary information

GFP 3 days	Laser after/Control after	Mann-Whitney	<0.0001	****	Yes
GFP 3 days	Laser 3 days after/Control 3 days after	Mann-Whitney	<0.0001	****	Yes
GFP 3 days	Laser before/Control before	Mann-Whitney	0.7251	ns	No
GFP 3 days	Control after/Control 3 days after	Wilcoxon matched-pairs signed rank	<0.0001	****	Yes
GFP 3 days	Control before/Control 3 days after	Wilcoxon matched-pairs signed rank	<0.0001	****	Yes
GFP 3 days	Laser before/ Laser after	Wilcoxon matched-pairs signed rank	<0.0001	****	Yes
GFP 3 days	Laser 3 days after/Laser after	Wilcoxon matched-pairs signed rank	<0.0001	****	Yes
GFP 3 days	Laser 3 days after/Laser before	Wilcoxon matched-pairs signed rank	<0.0001	****	Yes
GFP 3 days	Control after/ Control before	Wilcoxon matched-pairs signed rank	<0.0001	****	Yes
GFP 7 days	Laser before/Control before	Mann-Whitney	<0.0001	****	Yes
GFP 7 days	Laser 7 days after/Control 7 days after	Mann-Whitney	<0.0001	****	Yes
GFP 7 days	Laser after/Control after	Mann-Whitney	<0.0001	****	Yes
GFP 7 days	Laser 7 days after/Laser before	Wilcoxon matched-pairs signed rank	<0.0001	****	Yes
GFP 7 days	Laser 7 days after/Laser after	Wilcoxon matched-pairs signed rank	<0.0001	****	Yes
GFP 7 days	Laser before/ Laser after	Wilcoxon matched-pairs signed rank	<0.0001	****	Yes
GFP 7 days	Control before/Control 7 days after	Wilcoxon matched-pairs signed rank	<0.0001	****	Yes
GFP 7 days	Control after/Control 7 days after	Wilcoxon matched-pairs signed rank	<0.0001	****	Yes
GFP 7 days	Control after/ Control before	Wilcoxon matched-pairs signed rank	<0.0001	****	Yes

All collected data was assessed for normality and lognormality, and non-parametric tests, i.e. Mann–Whitney or Wilcoxon matched-pairs signed rank tests for different and for the same groups accordingly, were applied when distributions deviated from normality. The results of the statistical tests were indicated for each comparison. ***P*< 0.01, *****P*< 0.0001.

Disease Models & Mechanisms • Supplementary information

Table S2. Power analysis of size and GFP assessment of the spheroids before, after and at 3 days after laser treatment.

Para-meter	Compa-rison	Treat-ment	Time-point	Gr1 Name	Gr2 Name	Gr1 N	Gr2 N	Gr1 Mean	Gr2 Mean	Gr1 SD	Gr2 SD	Gr1 CI Low	Gr1 CI High	Gr2 CI Low	Gr2 CI High	Cohen's D	n Re-quired	Power	Adequate
GFP	*Laser vs Control*	*NA*	*before*	*Control*	*Laser*	*140*	*141*	*1*	*1*	*0.11*	*0.1*	*0.974*	*1.026*	*0.977*	*1.024*	*<0.01*	*261301*	*0.006*	*No*
GFP	*Laser vs Control*	*NA*	*after*	*Control*	*Laser*	*140*	*142*	*1.048*	*0.646*	*0.107*	*0.11*	*1.023*	*1.074*	*0.62*	*0.672*	*3.719*	*5*	*1*	*Yes*
GFP	*Laser vs Control*	*NA*	*3d*	*Control*	*Laser*	*139*	*137*	*0.952*	*0.754*	*0.108*	*0.155*	*0.926*	*0.978*	*0.717*	*0.792*	*1.478*	*14*	*1*	*Yes*
GFP	*before vs after*	*Control*	*NA*	*before*	*after*	*140*	*140*	*1*	*1.048*	*0.11*	*0.107*	*0.974*	*1.026*	*1.023*	*1.074*	*0.450*	*131*	*0.833*	*Yes*
GFP	*before vs 3d*	*Control*	*NA*	*before*	*3d*	*140*	*139*	*1*	*0.952*	*0.11*	*0.108*	*0.974*	*1.026*	*0.926*	*0.978*	*0.439*	*138*	*0.805*	*Yes*
GFP	*after vs 3d*	*Control*	*NA*	*after*	*3d*	*140*	*139*	*1.048*	*0.952*	*0.107*	*0.108*	*1.023*	*1.074*	*0.926*	*0.978*	*0.899*	*35*	*1*	*Yes*
GFP	*before vs after*	*Laser*	*NA*	*before*	*after*	*141*	*142*	*1*	*0.646*	*0.1*	*0.11*	*0.977*	*1.024*	*0.62*	*0.672*	*3.376*	*5*	*1*	*Yes*
GFP	*before vs 3d*	*Laser*	*NA*	*before*	*3d*	*141*	*137*	*1*	*0.754*	*0.1*	*0.155*	*0.977*	*1.024*	*0.717*	*0.792*	*1.882*	*10*	*1*	*Yes*
GFP	*after vs 3d*	*Laser*	*NA*	*after*	*3d*	*142*	*137*	*0.646*	*0.754*	*0.11*	*0.155*	*0.62*	*0.672*	*0.717*	*0.792*	*0.806*	*43*	*1*	*Yes*
Size	*Laser vs Control*	*NA*	*before*	*Control*	*Laser*	*140*	*141*	*1.002*	*0.998*	*0.097*	*0.111*	*0.979*	*1.025*	*0.972*	*1.024*	*0.035*	*21482*	*0.008*	*No*
Size	*Laser vs Control*	*NA*	*after*	*Control*	*Laser*	*140*	*142*	*0.97*	*0.962*	*0.11*	*0.106*	*0.944*	*0.996*	*0.937*	*0.987*	*0.077*	*4398*	*0.017*	*No*
Size	*Laser vs Control*	*NA*	*3d*	*Control*	*Laser*	*139*	*137*	*1.001*	*0.876*	*0.11*	*0.105*	*0.974*	*1.027*	*0.85*	*0.901*	*1.164*	*22*	*1*	*Yes*
Size	*before vs after*	*Control*	*NA*	*before*	*after*	*140*	*140*	*1.002*	*0.97*	*0.097*	*0.11*	*0.979*	*1.025*	*0.944*	*0.996*	*0.306*	*282*	*0.408*	*No*
Size	*before vs 3d*	*Control*	*NA*	*before*	*3d*	*140*	*139*	*1.002*	*1.001*	*0.097*	*0.11*	*0.979*	*1.025*	*0.974*	*1.027*	*0.013*	*166728*	*0.006*	*No*
Size	*after vs 3d*	*Control*	*NA*	*after*	*3d*	*140*	*139*	*0.97*	*1.001*	*0.11*	*0.11*	*0.944*	*0.996*	*0.974*	*1.027*	*0.277*	*343*	*0.315*	*No*
Size	*before vs after*	*Laser*	*NA*	*before*	*after*	*141*	*142*	*0.998*	*0.962*	*0.111*	*0.106*	*0.972*	*1.024*	*0.937*	*0.987*	*0.336*	*234*	*0.512*	*No*

Disease Models & Mechanisms • Supplementary information

Size	***before vs 3d***	***Laser***	***NA***	***before***	***3d***	***141***	***137***	***0.998***	***0.876***	***0.111***	***0.105***	***0.972***	***1.024***	***0.85***	***0.901***	***1.137***	***23***	***1***	***Yes***
Size	***after vs 3d***	***Laser***	***NA***	***after***	***3d***	***142***	***137***	***0.962***	***0.876***	***0.106***	***0.105***	***0.937***	***0.987***	***0.85***	***0.901***	***0.818***	***42***	***1***	***Yes***

Statistical power analysis for GFP intensity and spheroid size. Comprehensive pairwise comparisons between treatment groups (Laser vs. Control) and time points (before, after, 3d) are shown. Columns include: Parameter (GFP or Size), Comparison type, Treatment, Time point, Group 1/2 names, sample sizes (N), means, standard deviations (SD), 95% confidence intervals (CI low/high), Cohen's d effect size, required sample size for 80% power (n Required), achieved power (Power), and power adequacy (Yes/No). Bonferroni-Corrected α was set at 0.00556 (5% divided by 9 comparisons).

Disease Models & Mechanisms • Supplementary information

Table S3. Power analysis of size and GFP assessment of the spheroids before, after and at 7 days after laser treatment.

Parameter	Comparison	Treatment	Timepoint	Gr1 Name	Gr2 Name	Gr1 N	Gr2 N	Gr1 Mean	Gr2 Mean	Gr1 SD	Gr2 SD	Gr1 CI Low	Gr1 CI High	Gr2 CI Low	Gr2 CI High	Cohen's D	n Required	Power	Adequate
GFP	*Laser vs Control*	*NA*	*before*	*Control*	*Laser*	*135*	*132*	*1.023*	*0.973*	*0.079*	*0.113*	*1.004*	*1.043*	*0.945*	*1*	*0.521*	*99*	*0.924*	*Yes*
GFP	*Laser vs Control*	*NA*	*after*	*Control*	*Laser*	*139*	*138*	*1.056*	*0.61*	*0.112*	*0.172*	*1.029*	*1.083*	*0.569*	*0.651*	*3.073*	*5*	*1*	*Yes*
GFP	*Laser vs Control*	*NA*	*7d*	*Control*	*Laser*	*138*	*137*	*0.903*	*0.74*	*0.118*	*0.176*	*0.875*	*0.931*	*0.697*	*0.782*	*1.088*	*25*	*1*	*Yes*
GFP	*before vs after*	*Control*	*NA*	*before*	*after*	*135*	*139*	*1.023*	*1.056*	*0.079*	*0.112*	*1.004*	*1.043*	*1.029*	*1.083*	*0.339*	*230*	*0.496*	*No*
GFP	*before vs 7d*	*Control*	*NA*	*before*	*7d*	*135*	*138*	*1.023*	*0.903*	*0.079*	*0.118*	*1.004*	*1.043*	*0.875*	*0.931*	*1.197*	*21*	*1*	*Yes*
GFP	*after vs 7d*	*Control*	*NA*	*after*	*7d*	*139*	*138*	*1.056*	*0.903*	*0.112*	*0.118*	*1.029*	*1.083*	*0.875*	*0.931*	*1.330*	*17*	*1*	*Yes*
GFP	*before vs after*	*Laser*	*NA*	*before*	*after*	*132*	*138*	*0.973*	*0.61*	*0.113*	*0.172*	*0.945*	*1*	*0.569*	*0.651*	*2.491*	*7*	*1*	*Yes*
GFP	*before vs 7d*	*Laser*	*NA*	*before*	*7d*	*132*	*137*	*0.973*	*0.74*	*0.113*	*0.176*	*0.945*	*1*	*0.697*	*0.782*	*1.573*	*13*	*1*	*Yes*
GFP	*after vs 7d*	*Laser*	*NA*	*after*	*7d*	*138*	*137*	*0.61*	*0.74*	*0.172*	*0.176*	*0.569*	*0.651*	*0.697*	*0.782*	*0.744*	*50*	*1*	*Yes*
Size	*Laser vs Control*	*NA*	*before*	*Control*	*Laser*	*136*	*131*	*1.002*	*0.998*	*0.091*	*0.117*	*0.98*	*1.024*	*0.969*	*1.027*	*0.041*	*15215*	*0.008*	*No*
Size	*Laser vs Control*	*NA*	*after*	*Control*	*Laser*	*139*	*138*	*0.96*	*0.967*	*0.121*	*0.159*	*0.931*	*0.989*	*0.929*	*1.005*	*0.052*	*9536*	*0.01*	*No*
Size	*Laser vs Control*	*NA*	*7d*	*Control*	*Laser*	*138*	*137*	*1.016*	*0.85*	*0.121*	*0.152*	*0.987*	*1.045*	*0.814*	*0.887*	*1.209*	*20*	*1*	*Yes*
Size	*before vs after*	*Control*	*NA*	*before*	*after*	*136*	*139*	*1.002*	*0.96*	*0.091*	*0.121*	*0.98*	*1.024*	*0.931*	*0.989*	*0.396*	*169*	*0.682*	*No*
Size	*before vs 7d*	*Control*	*NA*	*before*	*7d*	*136*	*138*	*1.002*	*1.016*	*0.091*	*0.121*	*0.98*	*1.024*	*0.987*	*1.045*	*0.131*	*1516*	*0.045*	*No*
Size	*after vs 7d*	*Control*	*NA*	*after*	*7d*	*139*	*138*	*0.96*	*1.016*	*0.121*	*0.121*	*0.931*	*0.989*	*0.987*	*1.045*	*0.468*	*122*	*0.862*	*Yes*

Size	***before vs after***	***Laser***	***NA***	***before***	***after***	***131***	***138***	***0.998***	***0.967***	***0.117***	***0.159***	***0.969***	***1.027***	***0.929***	***1.005***	***0.220***	***543***	***0.157***	***No***
Size	***before vs 7d***	***Laser***	***NA***	***before***	***7d***	***131***	***137***	***0.998***	***0.85***	***0.117***	***0.152***	***0.969***	***1.027***	***0.814***	***0.887***	***1.087***	***25***	***1***	***Yes***
Size	***after vs 7d***	***Laser***	***NA***	***after***	***7d***	***138***	***137***	***0.967***	***0.85***	***0.159***	***0.152***	***0.929***	***1.005***	***0.814***	***0.887***	***0.752***	***49***	***1***	***Yes***

Statistical power analysis for GFP intensity and spheroid size. Comprehensive pairwise comparisons between treatment groups (Laser vs. Control) and time points (before, after, 7d) are shown. Columns include: Parameter (GFP or Size), Comparison type, Treatment, Time point, Group 1/2 names, sample sizes (N), means, standard deviations (SD), 95% confidence intervals (CI low/high), Cohen's d effect size, required sample size for 80% power (n Required), achieved power (Power), and power adequacy (Yes/No). Bonferroni-Corrected α was set at 0.00556 (5% divided by 9 comparisons); power was considered adequate at ≥0.80.

Disease Models & Mechanisms • Supplementary information

Table S4. Mixed-model statistics for marker expression across layers in whole-mount-stained spheroids.

Target	Time point	Contrast	Model	N	Omnibus	Omnibus_p	R2m	R2c	ICC	Effect (SE)	Test_stat	p_adj	Sig	Note
COL	3d	control_inner / laser_inner	G-GLMM	268	$\chi^2(7) = 70.385$	<0.001	0.2051	0.4723	0.3362	0.7294 (0.1006)	z = -2.287	0.0381	*	
COL	3d	control_middle / laser_middle	G-GLMM	268	$\chi^2(7) = 70.385$	<0.001	0.2051	0.4723	0.3362	0.6763 (0.0932)	z = -2.838	0.0182	*	
COL	3d	control_outer / laser_outer	G-GLMM	268	$\chi^2(7) = 70.385$	<0.001	0.2051	0.4723	0.3362	0.7581 (0.1045)	z = -2.008	0.0446	*	
COL	3d	control_sum / laser_sum	G-GLMM	268	$\chi^2(7) = 70.385$	<0.001	0.2051	0.4723	0.3362	0.7396 (0.1019)	z = -2.189	0.0381	*	
COL	7d	control_inner / laser_inner	G-GLMM	328	$\chi^2(7) = 35.023$	<0.001	0.0773	0.5609	0.5241	0.6973 (0.1243)	z = -2.023	0.172		
COL	7d	control_middle / laser_middle	G-GLMM	328	$\chi^2(7) = 35.023$	<0.001	0.0773	0.5609	0.5241	0.7695 (0.1355)	z = -1.489	0.182		
COL	7d	control_outer / laser_outer	G-GLMM	328	$\chi^2(7) = 35.023$	<0.001	0.0773	0.5609	0.5241	1.3078 (0.2283)	z = 1.537	0.182		
COL	7d	control_sum / laser_sum	G-GLMM	328	$\chi^2(7) = 35.023$	<0.001	0.0773	0.5609	0.5241	1.1564 (0.2021)	z = 0.831	0.406		
VIM	3d	control_inner / laser_inner	G-GLMM	124	$\chi^2(7) = 292.953$	<0.001	0.6416	0.7322	0.2527	0.7163 (0.1024)	z = -2.333	0.0393	*	
VIM	3d	control_middle / laser_middle	G-GLMM	124	$\chi^2(7) = 292.953$	<0.001	0.6416	0.7322	0.2527	0.6625 (0.0948)	z = -2.877	0.0161	*	
VIM	3d	control_outer / laser_outer	G-GLMM	124	$\chi^2(7) = 292.953$	<0.001	0.6416	0.7322	0.2527	0.9992 (0.1449)	z = -0.005	0.996		
VIM	3d	control_sum / laser_sum	G-GLMM	124	$\chi^2(7) = 292.953$	<0.001	0.6416	0.7322	0.2527	0.9447 (0.1364)	z = -0.394	0.925		
VIM	7d	control_inner / laser_inner	LN-LMM	104	$F(7, 91.1) = 29.175$	<0.001	0.6551	0.6727	0.0512	0.3274 (0.0819)	t(93.58) = -4.465	<0.001	***	
VIM	7d	control_middle / laser_middle	LN-LMM	104	$F(7, 91.1) = 29.175$	<0.001	0.6551	0.6727	0.0512	0.3116 (0.0779)	t(93.58) = -4.663	<0.001	***	
VIM	7d	control_outer / laser_outer	LN-LMM	104	$F(7, 91.1) = 29.175$	<0.001	0.6551	0.6727	0.0512	0.7871 (0.1968)	t(93.58) = -0.957	0.341		
VIM	7d	control_sum / laser_sum	LN-LMM	104	$F(7, 91.1) = 29.175$	<0.001	0.6551	0.6727	0.0512	0.6843 (0.1711)	t(93.58) = -1.517	0.177		
FIB	3d	control_inner / laser_inner	LN-LMM	96	$F(7, 83.8) = 12.976$	<0.001	0.3154	0.6728	0.5221	0.8642 (0.1358)	t(84.04) = -0.929	0.518		
FIB	3d	control_middle / laser_middle	LN-LMM	96	$F(7, 83.8) = 12.976$	<0.001	0.3154	0.6728	0.5221	0.7902 (0.1242)	t(84.04) = -1.498	0.518		

Disease Models & Mechanisms • Supplementary information

FIB	3d	control_outer / laser_outer	LN-LMM	96	F(7, 83.8) = 12.976	<0.001	0.3154	0.6728	0.5221	0.903 (0.1419)	t(84.04) = -0.649	0.518		
FIB	3d	control_sum / laser_sum	LN-LMM	96	F(7, 83.8) = 12.976	<0.001	0.3154	0.6728	0.5221	0.9014 (0.1417)	t(84.04) = -0.661	0.518		
FIB	7d	control_inner / laser_inner	LN-LMM	116	F(7, 102.4) = 12.161	<0.001	0.254	0.6579	0.5413	0.7638 (0.1457)	t(102.48) = -1.412	0.322		
FIB	7d	control_middle / laser_middle	LN-LMM	116	F(7, 102.4) = 12.161	<0.001	0.254	0.6579	0.5413	0.7052 (0.1345)	t(102.48) = -1.831	0.28		
FIB	7d	control_outer / laser_outer	LN-LMM	116	F(7, 102.4) = 12.161	<0.001	0.254	0.6579	0.5413	1.0333 (0.1971)	t(102.48) = 0.172	0.88		
FIB	7d	control_sum / laser_sum	LN-LMM	116	F(7, 102.4) = 12.161	<0.001	0.254	0.6579	0.5413	0.9716 (0.1854)	t(102.48) = -0.151	0.88		
Ki67	3d	control_inner - laser_inner	G-GLMM	256	F(7, 241.3) = 1.479	0.175	0.0222	0.4893	0.4777	1.9582 (0.7847)	t(242.05) = 2.496	0.053	.	a
Ki67	3d	control_middle - laser_middle	G-GLMM	256	F(7, 241.3) = 1.479	0.175	0.0222	0.4893	0.4777	0.9862 (0.7847)	t(242.05) = 1.257	0.286		a
Ki67	3d	control_outer - laser_outer	G-GLMM	256	F(7, 241.3) = 1.479	0.175	0.0222	0.4893	0.4777	0.8387 (0.7847)	t(242.05) = 1.069	0.286		a
Ki67	3d	control_sum - laser_sum	G-GLMM	256	F(7, 241.3) = 1.479	0.175	0.0222	0.4893	0.4777	0.9156 (0.7847)	t(242.05) = 1.167	0.286		a
Ki67	7d	control_inner - laser_inner	G-GLMM	253	F(7, 238.6) = 2.626	0.0125	0.0633	0.251	0.2004	-2.2168 (1.6518)	t(240.68) = -1.342	0.181		a
Ki67	7d	control_middle - laser_middle	G-GLMM	253	F(7, 238.6) = 2.626	0.0125	0.0633	0.251	0.2004	-4.467 (1.6518)	t(240.68) = -2.704	0.0293	*	a
Ki67	7d	control_outer - laser_outer	G-GLMM	253	F(7, 238.6) = 2.626	0.0125	0.0633	0.251	0.2004	-4.0266 (1.6518)	t(240.68) = -2.438	0.031	*	a
Ki67	7d	control_sum - laser_sum	G-GLMM	253	F(7, 238.6) = 2.626	0.0125	0.0633	0.251	0.2004	-3.6846 (1.6355)	t(240.7) = -2.253	0.0336	*	a

Statistical comparison of spatial differences in marker expression across layers (inner, middle, outer, and summed) of whole-mount-stained spheroids, for collagen I (COL), vimentin (VIM), fibronectin (FIB), and the proliferation marker Ki67, at 3 and 7 days post-ablation. Layer effects were assessed using linear or generalized linear mixed-effects models (Gaussian LMM (GA-LMM), Gamma GLMM (G-GLMM), or Log-normal LMM (LN-LMM)), with model family selected per marker via an evidence-based model-selection procedure rather than assumed a priori (see Methods). Only fixed slopes were used since random slopes led to singular fits. Target: the marker assessed. Timepoint: timepoint of sample

fixation. Contrast: the specific pair of layers compared. Model: the mixed-model family retained for inference for that marker. N: total number of complete case observations. Omnibus: the Type III test of the overall layer effect, reported as an F-test (Gaussian/Log-normal models) or a Wald χ^2 test (Gamma models), with degrees of freedom in parentheses. Omnibus_p: p-value for the omnibus test. R2m / R2c / ICC: marginal R^2 (variance explained by layer alone), conditional R^2 (variance explained by layer and random effects combined), and the intraclass correlation coefficient. Effect (SE): the estimated effect for the given layer contrast, reported as a ratio of means on the response scale (Gamma/Log-normal models) or a difference of means (Gaussian models), with its standard error in parentheses. Test_stat: the test statistic for the pairwise contrast, reported as a t-value (Gaussian/Log-normal models, with degrees of freedom) or a z-value (Gamma models, reflecting an asymptotic Wald test). p_adj: p-value for the layer contrast, adjusted for multiple comparisons within each marker (Benjamini-Hochberg FDR correction). Sig: significance code (***$P < 0.001$, *$P < 0.05$, ˙$P < 0.1$). Note: marker flagging model concerns (a= Only one structurally feasible candidate (fallback selection); Selected model's residual diagnostics indicate imperfect fit)

Table S5. Mixed-model statistics for marker expression upon treatment of whole-mount-stained spheroids.

Target	Con-trast	Model	N	Omnibus	Omni-bus_p	R2m	R2c	ICC	Effect (SE)	Test_stat	p_adj	Sig	Note
COL	Ctrl / Laser	LN-LMM	26	F(2, 22.7) = 3.924	0.0344	0.2384	0.2603	0.0288	1.0202 (0.255)	t(22.38) = 0.08	0.937		
COL	Ctrl / TGFB	LN-LMM	26	F(2, 22.7) = 3.924	0.0344	0.2384	0.2603	0.0288	0.565 (0.1386)	t(22.62) = -2.327	0.0615	.	
COL	Laser / TGFB	LN-LMM	26	F(2, 22.7) = 3.924	0.0344	0.2384	0.2603	0.0288	0.5538 (0.1508)	t(22.1) = -2.17	0.0615	.	
VIM	Ctrl / Laser	LN-LMM	40	F(2, 35.6) = 6.064	0.00541	0.2304	0.2714	0.0533	1.2964 (0.1889)	t(35.46) = 1.782	0.0883	.	
VIM	Ctrl / TGFB	LN-LMM	40	F(2, 35.6) = 6.064	0.00541	0.2304	0.2714	0.0533	0.7739 (0.1132)	t(35.66) = -1.752	0.0883	.	
VIM	Laser / TGFB	LN-LMM	40	F(2, 35.6) = 6.064	0.00541	0.2304	0.2714	0.0533	0.597 (0.0907)	t(36.31) = -3.395	0.00502	**	
FIB	Ctrl / Laser	LN-LMM	27	F(2, 22.2) = 8.453	0.00187	0.2911	0.5736	0.3985	0.6661 (0.0995)	t(22.11) = -2.721	0.0187	*	
FIB	Ctrl / TGFB	LN-LMM	27	F(2, 22.2) = 8.453	0.00187	0.2911	0.5736	0.3985	0.5731 (0.0818)	t(22.45) = -3.898	0.00225	**	
FIB	Laser / TGFB	LN-LMM	27	F(2, 22.2) = 8.453	0.00187	0.2911	0.5736	0.3985	0.8604 (0.1348)	t(22.2) = -0.96	0.348		
aSMA	Ctrl / Laser	LN-LMM	27	F(2, 22.2) = 6.3	0.0068	0.2086	0.5768	0.4653	2.2513 (0.7187)	t(22.09) = 2.542	0.0278	*	
aSMA	Ctrl / TGFB	LN-LMM	27	F(2, 22.2) = 6.3	0.0068	0.2086	0.5768	0.4653	0.7042 (0.2148)	t(22.36) = -1.15	0.262		
aSMA	Laser / TGFB	LN-LMM	27	F(2, 22.2) = 6.3	0.0068	0.2086	0.5768	0.4653	0.3128 (0.1047)	t(22.16) = -3.471	0.00647	**	
Ki67	Ctrl / Laser	G-GLMM	34	$\chi^2(2)$ = 2.416	0.299	0.0665	0.1713	0.1122	1.0424 (0.2352)	z = 0.184	0.854		
Ki67	Ctrl / TGFB	G-GLMM	34	$\chi^2(2)$ = 2.416	0.299	0.0665	0.1713	0.1122	1.3094 (0.2368)	z = 1.491	0.408		
Ki67	Laser / TGFB	G-GLMM	34	$\chi^2(2)$ = 2.416	0.299	0.0665	0.1713	0.1122	1.2562 (0.2846)	z = 1.006	0.471		

Disease Models & Mechanisms • Supplementary information

Statistical comparison of spatial differences in marker expression across treatments (Ctrl=Control, Laser= laser injured, TGFB= laser injured + TGF-β treated) of whole-mount-stained spheroids, for collagen I (COL), vimentin (VIM), fibronectin (FIB), α-smooth muscle actin (αSMA), and the proliferation marker Ki67, at 7 days post-ablation. Treatment effects were assessed using linear or generalized linear mixed-effects models (Gaussian LMM (GA-LMM), Gamma GLMM (G-GLMM), or Log-normal LMM (LN-LMM)), with model family selected per marker via an evidence-based model-selection procedure rather than assumed a priori (see Methods). Only fixed slopes were used since random slopes led to singular fits. Target: the marker assessed. Contrast: the specific pair of treatments compared. Model: the mixed-model family retained for inference for that marker. N: total number of complete case observations. Omnibus: the Type III test of the overall treatment effect, reported as an F-test (Gaussian/Log-normal models) or a Wald χ^2 test (Gamma models), with degrees of freedom in parentheses. Omnibus_p: p-value for the omnibus test. R2m / R2c / ICC: marginal R^2 (variance explained by treatment alone), conditional R^2 (variance explained by treatment and random effects (biological replicate) combined), and the intraclass correlation coefficient. Effect (SE): the estimated effect for the given treatment contrast, reported as a ratio of means on the response scale (Gamma/Log-normal models) or a difference of means (Gaussian models), with its standard error in parentheses. Test_stat: the test statistic for the pairwise contrast, reported as a t-value (Gaussian/Log-normal models, with degrees of freedom) or a z-value (Gamma models, reflecting an asymptotic Wald test). p_adj: p-value for the treatment contrast, adjusted for multiple comparisons within each marker (Benjamini-Hochberg FDR correction). Sig: significance code (**P< 0.01, *P< 0.05, ˙P < 0.1). Note: marker flagging model concerns.

Disease Models & Mechanisms • Supplementary information

Table S6. Differentially expressed proteins between control and lasered spheroids. Table showing differentially expressed proteins between Control *vs* Lasered spheroids evaluated with a moderated empirical Bayes test along with Benjamini–Hochberg post hoc test, Overrepresentation analysis (ORA) with upregulated or downregulated proteins using Gene Ontology Biological Pathways or Cellular Components.

Available for download at
https://journals.biologists.com/dmm/article-lookup/doi/10.1242/dmm.052855#supplementary-data

Table S7. Overrepresentation analysis (ORA) with upregulated proteins using Gene Ontology Biological Pathways.

Available for download at
https://journals.biologists.com/dmm/article-lookup/doi/10.1242/dmm.052855#supplementary-data

Table S8. Overrepresentation analysis (ORA) with downregulated proteins using Gene Ontology Biological Pathways.

Available for download at
https://journals.biologists.com/dmm/article-lookup/doi/10.1242/dmm.052855#supplementary-data

Table S9. Overrepresentation analysis (ORA) with upregulated proteins using Gene Ontology Cellular Components.

Available for download at
https://journals.biologists.com/dmm/article-lookup/doi/10.1242/dmm.052855#supplementary-data

Table S10. Overrepresentation analysis (ORA) with downregulated proteins using Gene Ontology Cellular Components.

Available for download at
https://journals.biologists.com/dmm/article-lookup/doi/10.1242/dmm.052855#supplementary-data

Table S11. Reagents and resources.

REAGENT/RESOURCE	SOURCE (company)	IDENTIFIER (article no)	Ab Dilution Factor
**Antibodies**			
**Primary Antibodies**			
mouse anti smooth muscle actin (αSMA), clone 1A4, RRID:AB_262054	Merck	SAB4200679	1:200
rabbit anti vimentin, RRID:AB_10003780	Novusbio	NBP1-31327	1:300
mouse anti myomesin, clone B4, RRID:AB_760349	Developmental Studies Hybridoma Bank, Iowa Univ. (DSHB)	mMaC B4	1:2
mouse anti collagen 1 α 1, clone SP1.D8, RRID:AB_528438	DSHB	SP1.D8	1:100
rabbit anti fibronectin, RRID:AB_2262874	Abcam	ab2413	1:200
rabbit anti Ki67 Rabbit (SP6), RRID:AB_10979488	Invitrogen	MA5-14520	1:300
mouse anti Ki67 (mouse), RRID:AB_2715512	Cell Signaling Technol.	9449S	1:100
chicken anti green fluorescent protein, RRID:AB_2534023	Invitrogen	A10262	1:100
mouse anti sarcomeric α-actinin, clone EA4, RRID:AB_2221571	Merck	7732	1:200
rabbit anti all actin, RRID:AB_476693	Merck	A2066	1:50

rabbit anti EH-myomesin	Gift by Irina Agarkova, ETHZ, (Agarkova et al., 2000)		1:200
rabbit anti connexin-43 (Cx43), RRID:AB_297976	Abcam	11370	1:100
rabbit anti myosin light chain 2v, RRID:AB_10645889	Synaptic Systems	310003	1:50
mouse anti myosin light chain 2a, RRID:AB_887737	Synaptic Systems	311011	1:200
rabbit anti Willms tumor protein (WT1), RRID:AB_2043201	Abcam	89901	1:100
mouse anti zona occludens protein-1 (ZO-1), RRID:AB_2533147	Invitrogen	33-9100	1:100
mouse anti DDR2, RRID:AB_1122158	Santa Cruz Biotech.	sc-81707	1:100
**Secondary Antibodies**			
goat anti-mouse IgG (H+L), Alexa Fluor™ 555, RRID:AB_2535844	Invitrogen	A21422	1:1000
Goat anti-rabbit IgG (H+L), Alexa Fluor™ 555, RRID:AB_2535849	Invitrogen	A21428	1:1000
goat anti-rabbit IgG (H+L), Alexa Fluor™ 633, RRID:AB_2535732	Invitrogen	A21071	1:1000
goat anti chicken IgY (H+L), Alexa Fluor™ 488, RRID:AB_2534096	Invitrogen	A11039	1:1000
goat anti rabbit IgG (H+L), Alexa Fluor™ 488, RRID:AB_143165	Invitrogen	A11008	1:1000
**Cell culture reagents and**			

Disease Models & Mechanisms • Supplementary information

**equipment**			
mTeSR1	Stem Cell Technologies	85850	
D-PBS,no Mg2+, no Ca 2+	Stem Cell Technologies	37350	
DMEM/F-12 with 15 mM HEPES	Stem Cell Technologies	36254	
DMEM +GlutaMAX +4.5g/l glucose	GIBCO	10566-016	
mFreSR	Stem Cell Technologies	5854	
ReLeSr	Stem Cell Technologies	5873	
STEMdiff™ CM Freezing Medium	Stem Cell Technologies	5030	
StemPro™ Accutase™	GIBCO	A1110501	
Antibiotic-Antimycotic	GIBCO	15240062	
Advanced DMEM/F12 medium	GIBCO	12634-028	
L-ascorbic acid-2-phosphate (AA2P)	Sigma	49752	
B-27™ Supplement, minus insulin	GIBCO	A1895601	
B-27™ Supplement (50X), serum-free	GIBCO	17504044	
Fetal Bovine Serum	GIBCO	26140079	
GlutaMAX	GIBCO	35050-061	
Gibco™ RPMI 1640 Medium	GIBCO	72400047	
TrypLE™ Select Enzyme (10X)	GIBCO	A1217701	
Sodium DL-lactate solution	SantaCruz Biotech.	sc-301818A	
Fibroblast Growth Medium 3 (FGM3)	Promo Cell/Merck	C23025	
CHIR99021 (GSK3 inhibitor)	Selleckchem	S2924	
ROCK inhibitor, Y27632 2 HCl	Selleckchem	S1049	
Wnt inhibitor, IWR-1-endo	Selleckchem	S7086	
Recombinant Human FGF2 Protein	R&D Systems	233-FB	
Retinoid acid	Sigma	R2625	
DMSO sterile filtered for cell culture	Sigma	D2438	
TGFβ recombinant human	Qkine	Qk010	

Disease Models & Mechanisms • Supplementary information

TGFβ inhibitor SB431542 (SB)	Tocris	1614	
LIVE/DEAD™ Viability/Cytotoxicity Kit	Invitrogen	L3224	
TUNEL Assay In situ cell death detection kit - TMR red	Roche/Merck	12156792910	
Caspase Assay CellEvent™	ThermoFisher	C10423	
Matrigel® hESC-qualified Matrix	Corning	354277	
Corning® Matrigel® Basement Membrane Matrix	Corning	354234	
**Cell lines**			
GM25256 (WTC-11)	Coriell Institute	GM25256	
WTC-mEGFP-TNNI1-cl172	Allen Inst./Coriell Institute	AICS-0037-172 iPSC	

All resources used in this study, including cell lines, antibodies, cell culture reagents, drugs, and kits.

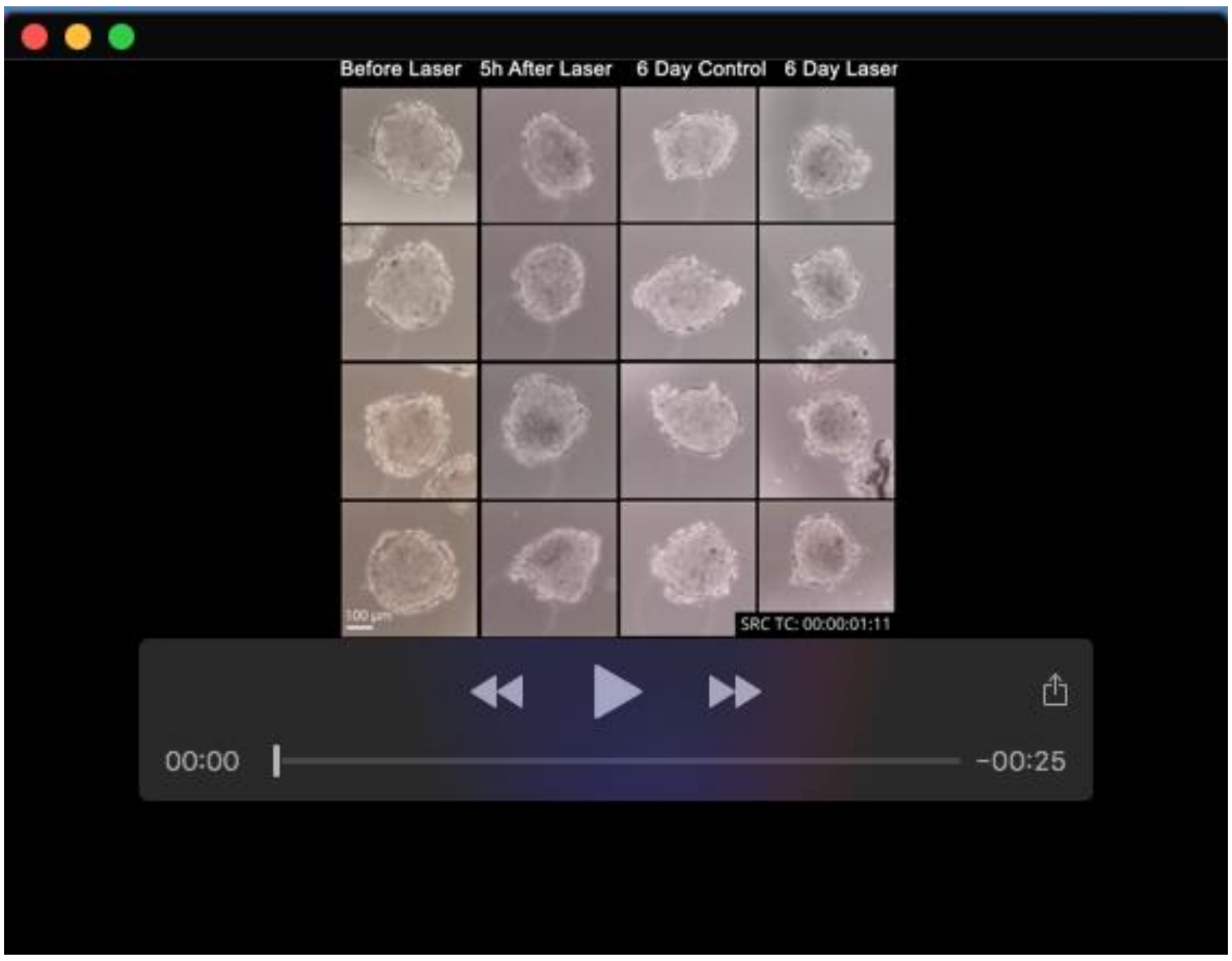

Movie 1. Spheroid contractions before and after laser injury. Shown are real-time videos of 4 representative examples from groups of spontaneously contracting spheroids at different time points.

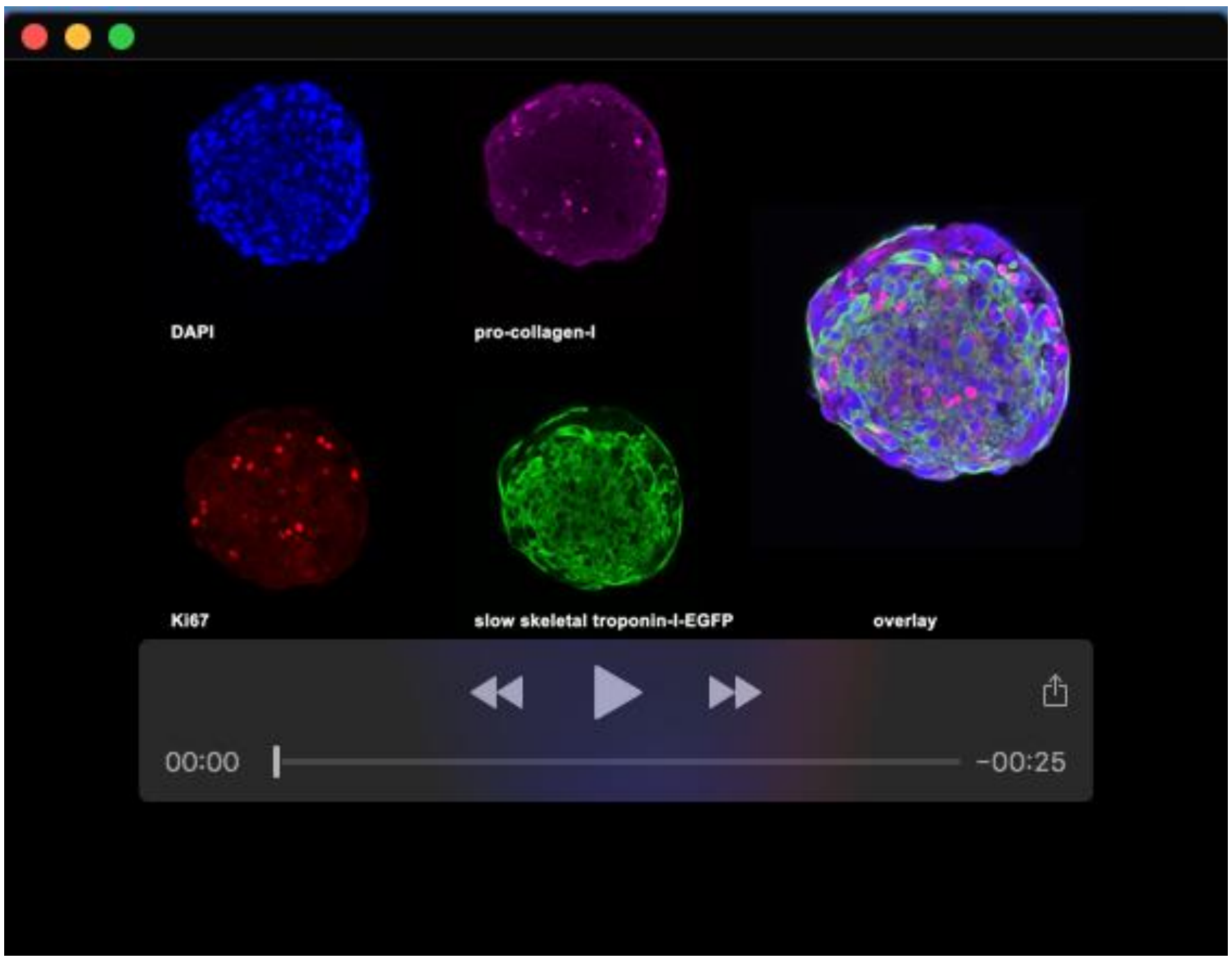

Movie 2. Moving through a confocal image stack of an uninjured, cleared and immunostained spheroid. The focus is moving through the recorded images in the stack showing the separated immunostained channels.

Disease Models & Mechanisms • Supplementary information

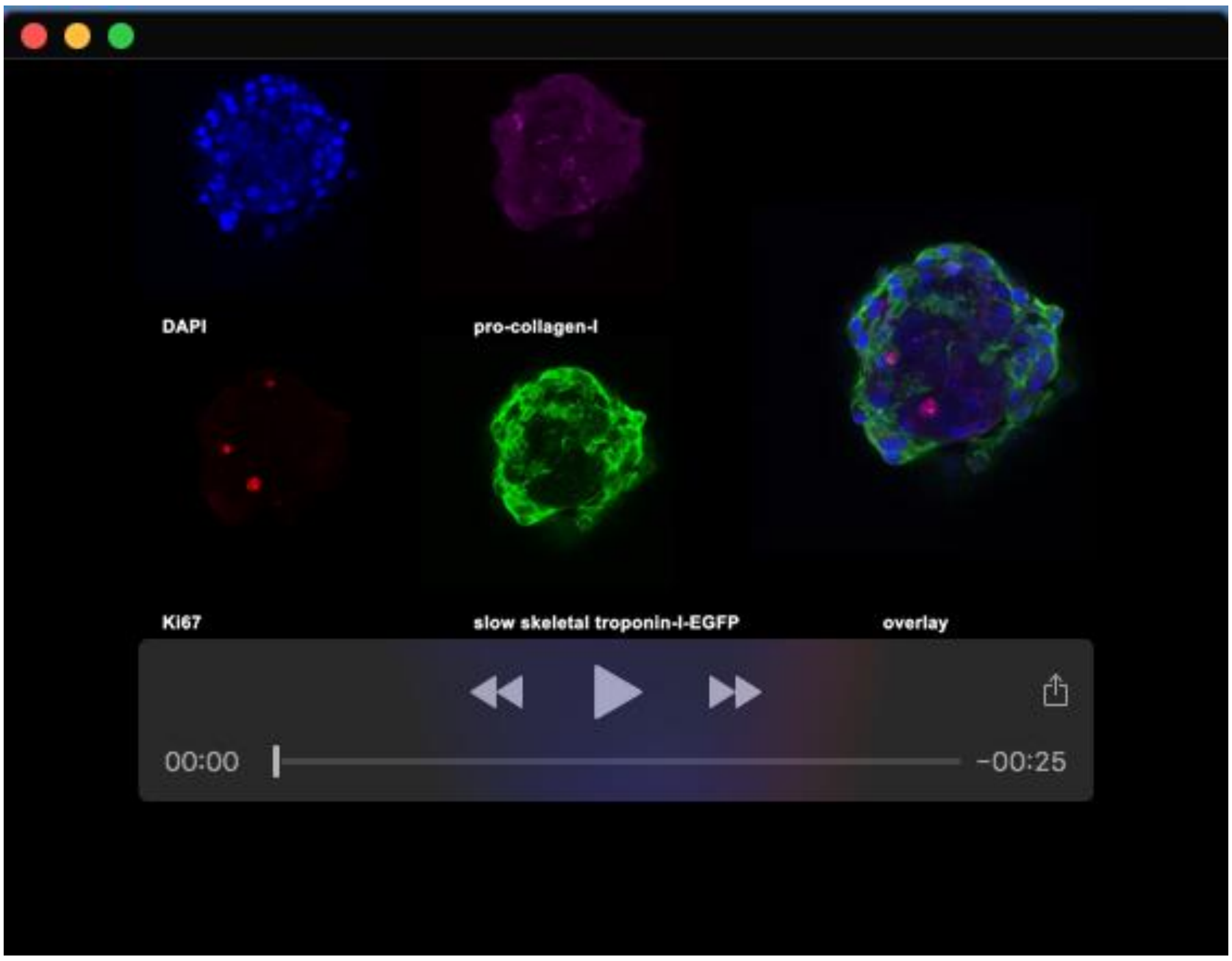

Movie 3. Moving through a confocal image stack of a cleared and immunostained spheroid 3 days after laser injury. The focus is moving through the recorded images in the stack showing the separated immunostained channels.
